# Environmentally Induced Transgenerational Epigenetic Reprogramming of Primordial Germ Cells and the Subsequent Germ Line

**DOI:** 10.1371/journal.pone.0066318

**Published:** 2013-07-15

**Authors:** Michael K. Skinner, Carlos Guerrero-Bosagna M. Haque, Eric Nilsson, Ramji Bhandari, John R. McCarrey

**Affiliations:** 1 Center for Reproductive Biology, School of Biological Sciences, Washington State University, Pullman, Washington, United States of America; 2 Department of Biology, University of Texas at San Antonio, San Antonio, Texas, United States of America; Baylor College of Medicine, United States of America

## Abstract

A number of environmental factors (e.g. toxicants) have been shown to promote the epigenetic transgenerational inheritance of disease and phenotypic variation. Transgenerational inheritance requires the germline transmission of altered epigenetic information between generations in the absence of direct environmental exposures. The primary periods for epigenetic programming of the germ line are those associated with primordial germ cell development and subsequent fetal germline development. The current study examined the actions of an agricultural fungicide vinclozolin on gestating female (F0 generation) progeny in regards to the primordial germ cell (PGC) epigenetic reprogramming of the F3 generation (i.e. great-grandchildren). The F3 generation germline transcriptome and epigenome (DNA methylation) were altered transgenerationally. Interestingly, disruptions in DNA methylation patterns and altered transcriptomes were distinct between germ cells at the onset of gonadal sex determination at embryonic day 13 (E13) and after cord formation in the testis at embryonic day 16 (E16). A larger number of DNA methylation abnormalities (epimutations) and transcriptional alterations were observed in the E13 germ cells than in the E16 germ cells. These observations indicate that altered transgenerational epigenetic reprogramming and function of the male germline is a component of vinclozolin induced epigenetic transgenerational inheritance of disease. Insights into the molecular control of germline transmitted epigenetic inheritance are provided.

## Introduction

Environmentally induced epigenetic transgenerational inheritance of disease and phenotypic variation involves the germline transmission of altered epigenetic information in the absence of direct exposure [1], [2]. The critical window for exposure is during the period of epigenetic reprogramming of the developing germ line coincident with the onset of fetal gonadal sex determination [1], [2], [3]. The primordial germ cells (PGCs) undergo an erasure of DNA methylation during migration to the genital ridge and colonization of the fetal gonads and then the germline genome initiates remethylation of DNA at the onset of gonadal sex determination in a sex specific manner [4], [5]. Previous research demonstrated that exposure of an F0 generation gestating female to the agricultural fungicide vinclozolin during PGC development in the developing fetuses promotes epigenetic transgenerational inheritance of disease [1], [3] and epigenetic alterations in the F3 generation descendants [1], [6]. Subsequently, a number of different environmental toxicants have been shown to promote exposure specific alterations in the F3 generation sperm epigenome (DNA methylation) [7]. These include dioxin [8], [9], a plastic mixture (bisphenol A (BPA) and phthalates) [10], [11], [12], the pesticide methoxychlor [1], a pesticide and insecticide mixture (permethrin and DEET) [13], and a hydrocarbon mixture (JP8 jet fuel) [14]. In addition to environmental toxicants, nutrition [15], [16] and stress [17], [18] can promote epigenetic transgenerational phenotypes. The primary site of action of these different environmental factors must be in the germ line in order to promote epigenetic transgenerational inheritance. This phenomenon has been demonstrated in a wide variety of species including rats [1], [3], humans [19], [20], mice [9], [21], plants [22], [23], worms [24], [25], and flies [26], [27]. The current study used an outbred rat model [1] and the agricultural fungicide vinclozolin [28] to promote the epigenetic transgenerational inheritance of abnormalities that include testis spermatogenic defects and male infertility [1], [29], prostate disease [3], [30], kidney disease [3], behavior alterations (e.g. anxiety) [18], [31], [32], mammary gland tumor development [3], immune abnormalities [3], and ovarian disease [7], [33].

The molecular mechanism starts with the induction of an epigenetic alteration in the developing male germ line during fetal gonadal sex determination that promotes a permanent alteration in the germline epigenome (e.g. sperm) [1], [2], [6]. The germ line then transmits this altered epigenome to the ensuing embryo, which then leads to all tissues and cell types having altered transgenerational transcriptomes and epigenomes that can be associated with adult onset disease [2], [34], [35]. The altered germline epigenome appears to be imprinted-like in that it escapes the normal erasure of DNA methylation following fertilization to transmit the epigenome transgenerationally in a parent-of-origin (male) specific manner [2], [36]. The current study was designed to investigate the transgenerational effects on the F3 generation germ line to determine if these cells maintain altered developmental programming of the epigenome and transcriptome.

Germ cell development is initiated in mammals when primordial germ cells (PGCs) are derived from the epiblast during embryonic development and subsequently migrate to the developing genital ridges [37], [38], [39]. The PGCs then colonize the indifferent gonads prior to gonadal sex determination shortly before the initiation of differentiation into the male or female germ line depending on the sex of the fetus [39]. After several mitotic events in the developing ovary the female germ cells enter prophase 1 of meiosis and form nests of primary oocytes that then develop after birth (rodents) into primordial follicles [40]. In the developing testis the germ cells continue to proliferate and organize into the developing cords that will eventually develop into seminiferous tubules at the onset of puberty [41]. As PGCs enter the developing gonads DNA methylation is largely erased and several days later global de novo methylation occurs to re-establish the methylome in these cells. Certain regions of the genome (e.g. imprinted genes) adopt sex-specific DNA methylation patterns at this time [4], [5]. In the fetal testis, the germ cells continue to proliferate mitotically and then enter a mitotic arrest near birth and resume proliferation a few days after birth in the rodent [42], [43]. At the onset of puberty the spermatogonia develop at the basal surface of the seminiferous tubule and become the stem cell for the spermatogenic process. The spermatogenic process involves mitotic divisions leading to development of spermatocytes that then enter meiosis to give rise to haploid spermatids which differentiate into spermatozoa that are released into the lumen of the tubule [44]. The final stage of maturation occurs in the epididymis when the sperm develop the capacity for motility [45]. Epigenetic alterations in DNA methylation have been described during the spermatogenic and maturation processes to facilitate subsequent developmental events.

The supporting somatic cell in the fetal gonad is the precursor Sertoli cell that in the adult forms the seminiferous tubule and provides physical support for the male germ cells [46]. The interstitial cells include the Leydig cells in both the fetal and adult testis. Factors that promote the epigenetic transgenerational inheritance of disease prior to and during fetal gonadal sex determination can involve direct actions on the PGCs or subsequent germ cells, as well as indirect actions on the somatic cells such as precursor Sertoli cells and Leydig cells that subsequently influence the germ cells. Although the direct versus indirect actions of the environmental factors *in vivo* cannot be distinguished, the ultimate target cell required to facilitate transgenerational inheritance must be the germ cells.

The experimental design used isolated PGCs from testes at embryonic day 13 (E13) that correspond to the initiation of testis development and a stage at which DNA methylation has been erased from the germ cell genome. In addition, type T1 prospermatogonia were isolated from E16 testes at a stage following cord formation when remethylation of the germline genome has begun [47]. The current study examined the transcriptome and epigenome (DNA methylation) in germ cells from these two stages of development in F3 generation control and vinclozolin lineage transgenerational animals. The objective was to obtain insights into germline epigenetic programming and its role in the epigenetic transgenerational inheritance phenomenon.

## Results

The experimental design involved the exposure of an F0 generation gestating female outbred Spague-Dawley rat during the period of embryonic days 8–14 that correlate with the end of primordial germ cell (PGC) migration and early events of gonadal sex determination [1], [2]. The F0 generation females were exposed to a vehicle (dimethylsulfoxide DMSO) as control or to vinclozolin, as described in the Methods. The F1 generation offspring were bred to generate the F2 generation and the F2 generation offspring were bred to generate the F3 generation offspring, as previously described [3]. No sibling or cousin crosses were used to avoid any inbreeding artifacts. The timed pregnant F2 generation females were used to isolate the F3 generation control and vinclozolin lineage fetal gonads at the E13 and E16 time points. The F3 generation E13 PGC and E16 prospermatogonia were isolated as described in the Methods and found to be ≥85% pure based on morphological criteria [48]. RNA and DNA were isolated from the freshly isolated cells to examine gene expression by microarray analysis and DNA methylation by methylated DNA immunoprecipitation (MeDIP) followed by analysis on a genome-wide promoter tiling array (Chip) using a comparative hybridization MeDIP-Chip analysis between control and vinclozolin lineage samples [6] as described in Methods. This allowed a comparison of the transcriptome or epigenome alterations in F3 vinclozolin lineage germ cells at E13 and E16. Three separate experiments involving different sets of animals and different germ cell isolations were analyzed with three different microarray and MeDIP-Chip analyses.

**Figure 1:**
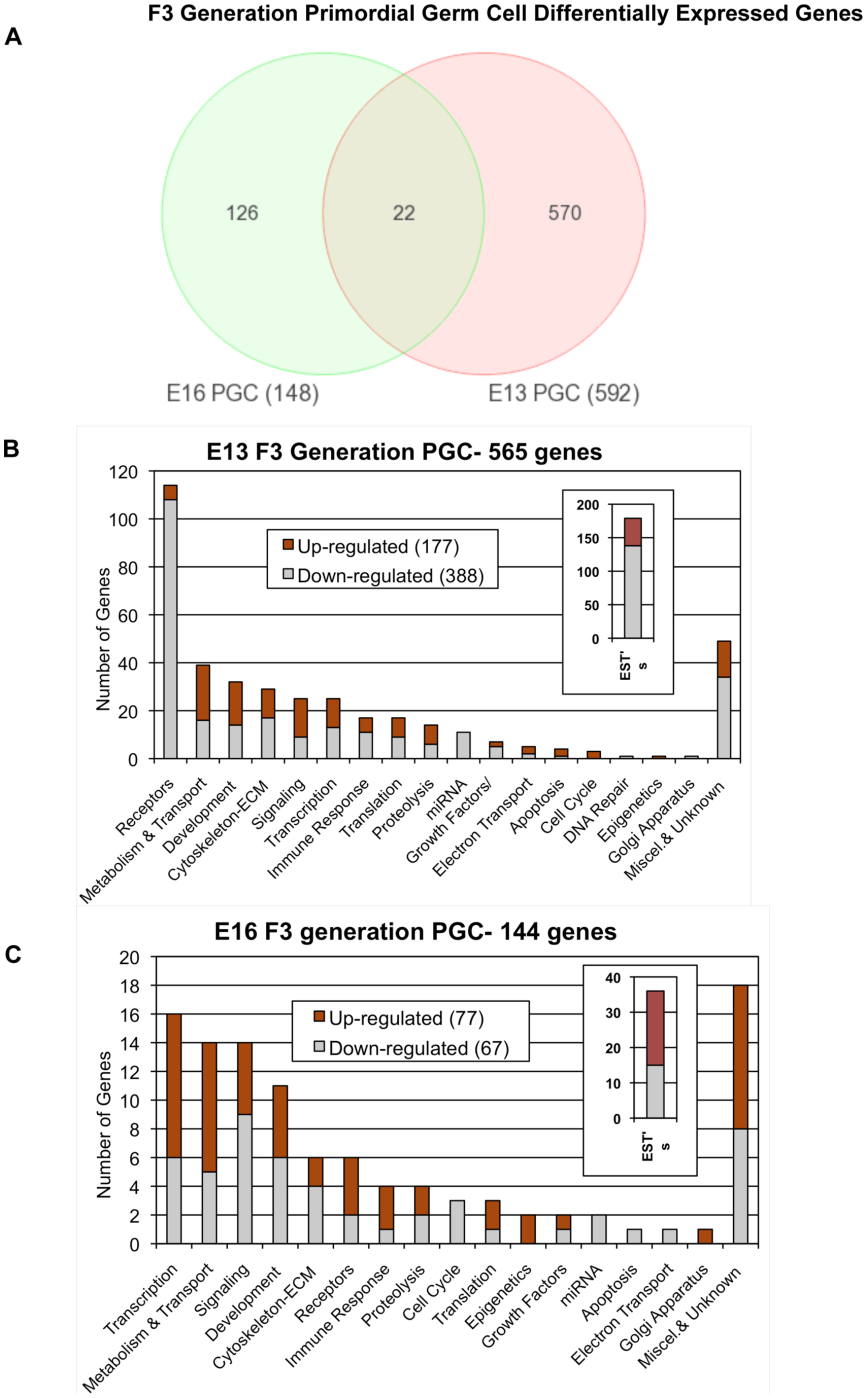
Genes with mRNA expression levels significantly different between control and vinclozolin lineage F3 generation germ cells at E13 and E16. (A) Number of differentially expressed genes unique to E13 PGCs, unique to E16 prospermatogonia, and common to both. (B and C) Numbers of differentially expressed genes in germ cells categorized by function for; (B) E13 and (C) E16.

The germ cell transcriptome analyses demonstrated that all arrays were of good quality with no abnormal hybridization detected, Figure S1. Differential gene expression between control and vinclozolin lineage F3 generation germ cells was determined as previously described [34]. There were 592 differentially expressed genes in germ cells at E13 and 148 differentially expressed genes in germ cells at E16, Figure 1A. The complete lists of differentially expressed genes are presented in Tables S1 and S2. Interestingly, comparison of the gene sets between E13 and E16 identified only 25 genes common to both lists. Observations demonstrate the majority of differentially expressed genes are distinct between these two developmental stages, Figure 1A and Tables S1 and S2. The differentially expressed genes identified at each stage were clustered into functional categories as presented in Figure 1. Observations demonstrated the E13 and E16 gene sets generally had similar gene categories represented for both the up and down regulated genes. Although the specific E13 and E16 germ cell differentially expressed gene sets are primarily distinct, overlap was observed in major functional gene categories affected. The specific gene category for each gene is presented in Tables S1 and S2.

**Figure 2:**
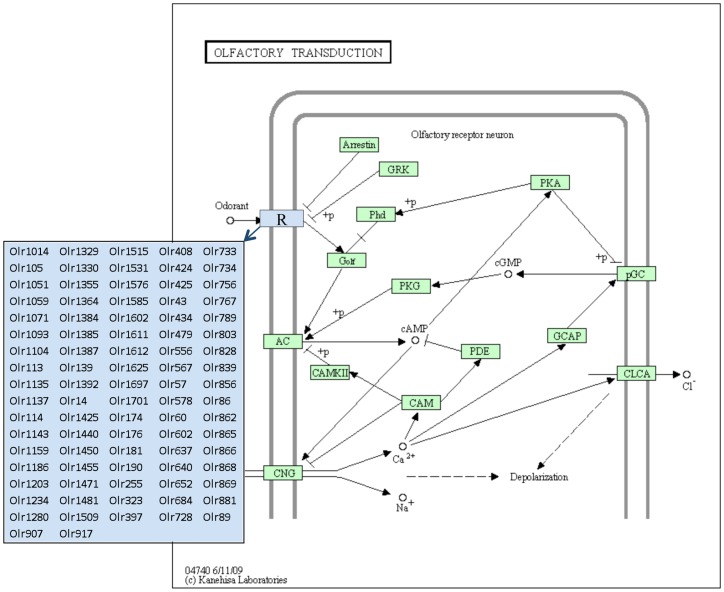
Olfactory Transduction Pathway showing olfactory receptor genes differentially expressed between F3 generation E13 PGC vinclozolin and control lineage rats. Adapted from Kyoto Encyclopedia of Genes and Genomes pathway rno04740.

The E13 and E16 germ cells differentially expressed gene sets were analyzed for specific cellular pathways and processes as previously described [34], see Methods. A list of cellular pathways and processes that have three or more genes from the gene sets is presented in Table 1. Interestingly, 24 different pathways were identified for the E13 differential gene expression set, but only one pathway had three or more genes for the E16 gene set. Therefore, the genes in the E16 list were more widely distributed and not enriched for specific pathways, while the E13 gene set did show enriched participation in specific pathways, Table 1. A unique affected pathway identified from the E13 differentially expressed gene set was the olfactory transduction pathway, which had 64 different genes enriched in this pathway. As shown in Figure 2, all of these genes were olfactory receptors that have been shown to require critical epigenetic regulation [49], [50].

**Table 1:** Pathways enriched with F3 generation E13 and E16 germ cell gene lists.

Pathways Enriched with F3 generation E13 PGC gene lists	
Pathway Name	# genes affected
Olfactory transduction	64
Autoimmune thyroid disease	6
Measles	6
Systemic lupus erythematosus	6
Transcriptional misregulation in cancer	6
Allograft rejection	5
Calcium signaling pathway	5
Intestinal immune network for IgA production	5
Rheumatoid arthritis	5
Staphylococcus aureus infection	5
Viral myocarditis	5
Asthma	4
Neuroactive ligand-receptor interaction	4
Amoebiasis	3
Cell adhesion molecules (CAMs)	3
Fc gamma R-mediated phagocytosis	3
Glycolysis/Gluconeogenesis	3
Graft-versus-host disease	3
HTLV-I infection	3
Influenza A	3
Natural killer cell mediated cytotoxicity	3
Regulation of actin cytoskeleton	3
Tuberculosis	3
Type I diabetes mellitus	3

Pathways Enriched with F3 generation E16 germline gene list	
Glutamatergic synapse	3

**Figure 3:**
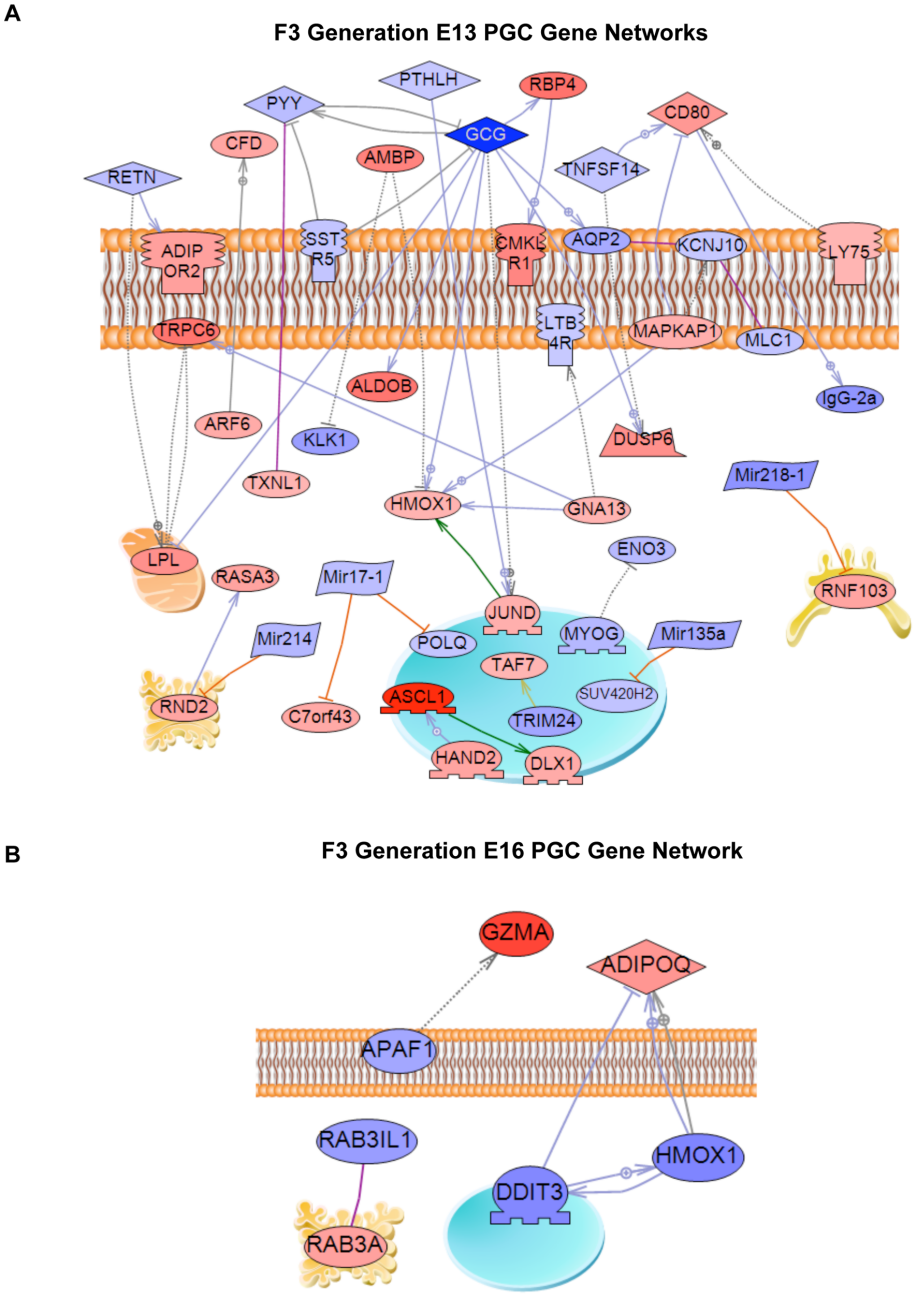
Gene network of known relationships among those genes differentially expressed in control- compared to vinclozolin lineage F3 generation germ cells. (A) E13 network, (B) E16 network. Gene node shape code: oval and circle – protein; diamond – ligand; irregular polygon – phosphatase; circle/oval on tripod platform – transcription factor; ice cream cone – receptor. Red colored nodes are up-regulated genes, blue color are down-regulated genes. Grey connecters represent general regulation, blue – expression regulation, purple – binding, green – promoter binding, orange – microRNA effect. Cell membrane, nucleus, mitochondria, endoplasmic reticulum and golgi localizations are indicated. Network was derived using Pathway Studio™ software.

A final analysis of the E13 and E16 differentially expressed genes identified a gene network based on previous literature involving binding and functional interactions between the various genes in the specific gene sets, see Methods. A gene network for the E13 gene set is shown in Figure 3A and identifies a number of extracellular regulators, signaling molecules and transcription factors that integrate functionally. In contrast, the E16 differential expression list generated a small network with only six genes, Figure 3B. Vinclozolin was found to induce altered transgenerational germline transcriptomes that are distinct in PGCs and prospermatogonia.

**Figure 4:**
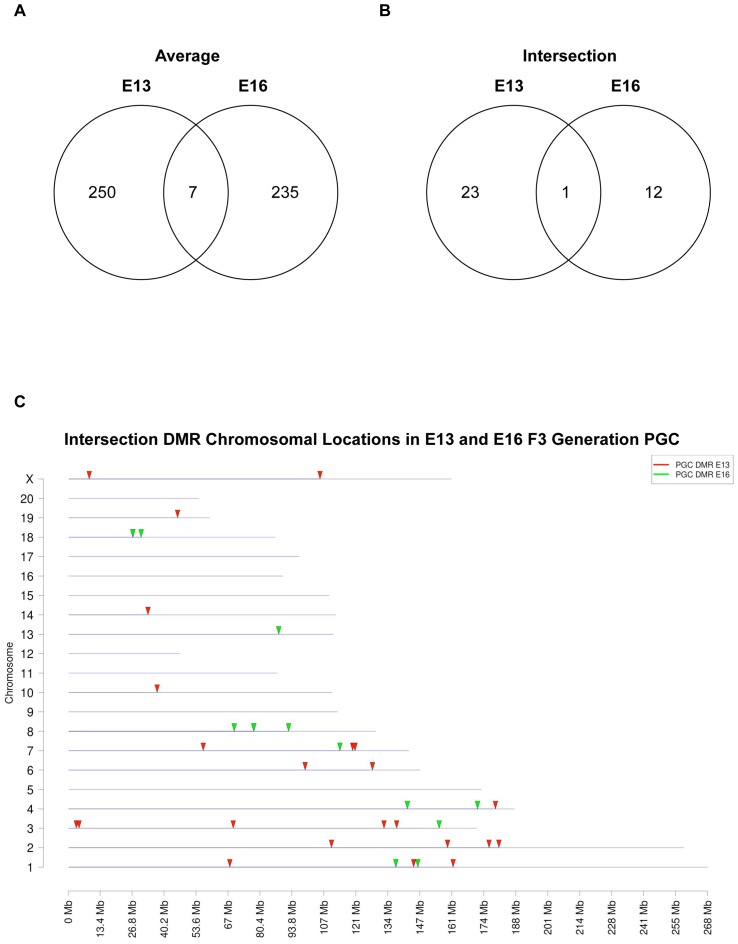
Number of vinclozolin induced transgenerational DMR detected in F3 generation E13 and E16 germ cells, using two different bioinformatic analyses. (A) The analysis is performed by averaging data from three comparative MeDIP-Chip per developmental time using a statistical cut-off of p<10^−4^. (B) The analysis is performed by selecting only the DMR that repeatedly appeared as significantly changed in all MeDIP-Chip comparisons (intersection) using a cut-off of p<10^−4^. (C) A graphical representation of the DMR location in all chromosomes in the rat that were obtained through the intersection analysis for both E13 and E16 germ cell DMR.

Genomic DNA from the E13 and E16 germ cells was isolated and used to identify altered differential DNA methylation regions (DMR) (epimutations) between the F3 generation control versus vinclozolin lineage germ cells with MeDIP-Chip analyses. One approach used in this type of analysis is to take the data from three different experiments and generate an average mean to assess statistical significance. This approach generated 257 DMR for the E13 PGCs and 242 DMR for the E16 germ cells with a statistically significant difference (p<10^−4^), Figure 4A. These DMR are the result of what is termed an “average” analysis. In contrast a second approach, previously used by our laboratory, used a more stringent analysis requiring a reproducible and statistically significant (p<10^−4^) epimutation (i.e. DMR) to be present in each separate experiment. This is termed an “intersection” analysis. This intersection analysis identified 24 DMR for E13 PGCs and 13 DMR for E16 germ cells, Figure 4B and Table 2. Interestingly, very few of the DMR overlapped between the E13 and E16 germ cell datasets. The intersection DMR had one overlapped promoter gene, Figure 4, identified as *Pigb*, Table 2. The average DMR datasets had seven overlapped gene promoter in both E13 and E16 germ cells. These were identified as *Pigb, Hmx2, Trx3, LOC499585, H1f0, Pim3* and *Nign3*, Table 2. Therefore, as observed with the differential gene expression datasets, Figure 1, the differential DNA methylation regions (DMR) also had negligible overlap between the E13 and E16 germ cell samples. Interestingly, a comparison with mature sperm epimutations previously identified [6] demonstrated no overlap with the E13 or E16 germ cell epimutations. The chromosomal locations of the intersection DMR for E13 and E16 germ cells are presented in Figure 4C. The majority of the autosomes and the X chromosome contained one or more DMR.

**Table 2:** DMR from (A) E13 PGC and (B) E16 germ cells.

A. E13 PGC DMR							
Gene Symbol	Description	Entrez gene ID	Significance (p< = )	Chr	Start	End	Region size(bp)
Adsl	adenylosuccinate lyase	315150	3.78655048468349e-14	7	119221149	119221875	726
Arfip1	ADP-ribosylation factor interacting protein 1	60382	1.25959624484326e-08	2	176355108	176356091	983
Arl9	ADP-ribosylation factor-like 9	289565	3.51696997190646e-16	14	33460390	33461186	796
Bglap	bone gamma-carboxyglutamate (gla) protein	25295	1.52971909876422e-11	2	180484919	180487344	2425
Cst3	cystatin C	25307	1.9676677569316e-05	3	137654243	137654843	600
Dstn	destrin	502674	1.8037968573233e-17	3	132358362	132359174	812
Ift80	intraflagellar transport 80 homolog (Chlamydomonas)	295106	4.29141490491877e-06	2	158985833	158986919	1086
Il13ra1	interleukin 13 receptor, alpha 1	252963	1.64249772706228e-06	X	8820511	8821111	600
LOC499584	LRRGT00202	499584	1.89613932385601e-09	2	110357837	110358437	600
Lyz2	lysozyme 2	25211	1.07459600381755e-07	7	56612889	56613489	600
Maf	v-maf musculoaponeurotic fibrosarcoma oncogene homolog (avian)	54267	2.18687594147628e-06	19	45861256	45861856	600
Ndor1	NADPH dependent diflavin oxidoreductase 1	311799	1.00275312476137e-12	3	3420722	3421807	1085
Olr111	olfactory receptor 111	405006	3.29398302124425e-09	1	161319052	161319652	600
Olr469	olfactory receptor 469	295739	6.29429665927461e-07	3	69170933	69171533	600
Pigb	phosphatidylinositol glycan anchor biosynthesis, class B	315807	6.9712710596944e-46	8	77794848	77795448	600
Prima1	proline rich membrane anchor 1	690195	2.11444223671492e-08	6	127523443	127524268	825
Rab38	RAB38, member RAS oncogene family	252916	6.93621121098414e-07	1	144784135	144785018	883
Sdccag3	serologically defined colon cancer antigen 3	306322	5.6108175079916e-06	3	4558626	4559226	600
Sec24a	SEC24 family, member A (S. cerevisiae)	287275	2.18834047032063e-07	10	37281198	37281798	600
Slco1b3	solute carrier organic anion transporter family, member 1b3	58978	5.94204976416307e-11	4	179042204	179042804	600
Snrk	SNF related kinase	170837	4.01338399294972e-07	X	105537519	105538210	691
Sptb	spectrin, beta, erythrocytic	314251	1.24562330248275e-06	6	99316967	99317667	700
Tob2	transducer of ERBB2, 2	315159	1.75285493754996e-06	7	120210412	120211293	881
Ube2s	ubiquitin-conjugating enzyme E2S	292588	1.1890299638469e-06	1	67742366	67742966	600

B. E16 Germ Cell DMR							
Gene Symbol	Description	Entrez gene ID	Significance (p< = )	Chr	Start	End	Region size(bp)
Atp1a4	ATPase, Na+/K+ transporting, alpha 4 polypeptide	29132	1.28994330234613e-06	13	88248651	88249535	884
Brd8	bromodomain containing 8	291691	2.44741320993066e-06	18	27098792	27099576	784
Crbn	cereblon	297498	1.22510462678683e-06	4	142153894	142154908	1014
Eef1d	eukaryotic translation elongation factor 1 delta (guanine nucleotide exchange protein)	300033	1.29320375181597e-05	7	113878232	113878832	600
Kif20a	kinesin family member 20A	361308	1.90112286587884e-08	18	27098792	27099866	1074
LOC293103	similar to RIKEN cDNA 0610007P06	293103	7.00735060708582e-06	1	146544171	146545251	1080
Loh12cr1	loss of heterozygosity, 12, chromosomal region 1 homolog (human)	362452	1.92570356090438e-09	4	171545884	171547070	1186
Pcdhga2	protocadherin gamma subfamily A, 2	498846	1.33187497734634e-06	18	30576558	30577248	690
Pdcd7	programmed cell death 7	363082	3.79409190332191e-09	8	69589977	69590664	687
Pigb	phosphatidylinositol glycan anchor biosynthesis, class B	315807	1.12719999943101e-42	8	77794848	77795448	600
RGD1309873	similar to hypothetical protein BC010003	363112	8.33745272768619e-07	8	92384988	92385588	600
Sdc4	syndecan 4	24771	1.01771870918723e-16	3	155467470	155468562	1092
Slc28a1	solute carrier family 28 (sodium-coupled nucleoside transporter), member 1	116642	2.14393247942146e-07	1	137320526	137321526	1000

**Figure 5:**
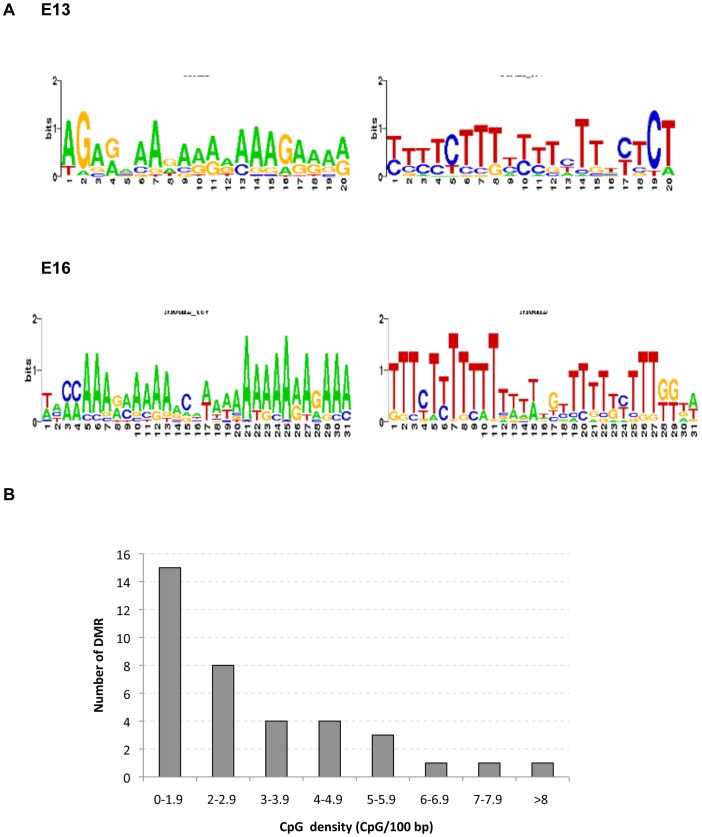
Genomic features of the DMR identified. (A) The forward and reverse sequence motifs obtained with the MEME suite tool GLAM2 for the vinclozolin induced transgenerational DMR from E13 and E16 F3 generation germ cells. (B) Shows the distribution of CpG sites (CpG/100bp) in vinclozolin induced transgenerational DMR obtained from both the E13 and E16 germ cells.

The intersection DMR for both E13 and E16 germ cells were next investigated for potential genomic features associated with the genomic regions of each DMR, as previously described [6], [7]. Common DNA sequence motifs identified in 100% of the intersection DMR are shown in Figure 5A for both forward and reverse strands of DNA. These motifs are similar to an environmentally induced differential DNA methylation region motif 1 (EDM1) previously associated with vinclozolin induced transgenerational sperm DMR in the F3 generation [6]. The A rich motif is similar to those known to associate with high mobility group (HMG) box proteins that bind and bend DNA [51]. Another genomic feature investigated involves the CpG density (CpG/100 bp) within each DMR. Interestingly, all the intersection DMR had less than 10 CpG/100 bp, with the majority being only 1–2 CpG/100 bp, Figure 5B. Previous studies have demonstrated that environmentally induced DMR in F3 generation sperm have a low CpG density of <15 CpG/100 bp [6], [7]. Therefore, the transgenerational E13 and E16 germ cell DMR have similar genomic features as sperm DMR of a sequence motif EDM1 and low density CpG density.

**Figure 6:**
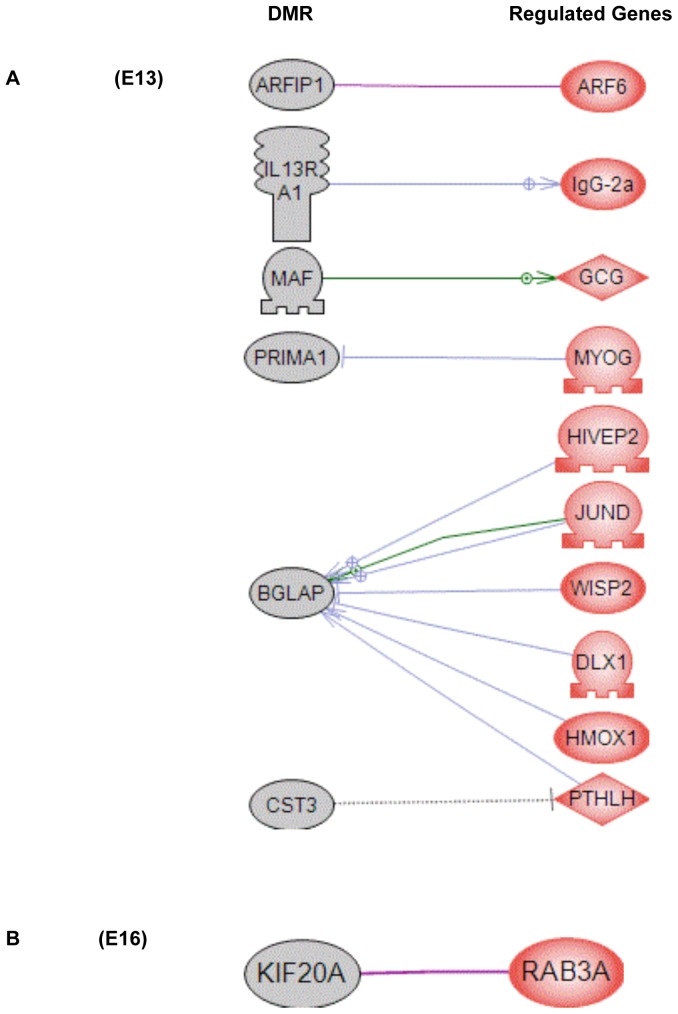
Known relationships between genes having DMR in their promoter regions (grey nodes) and differentially expressed genes (red nodes) in control- compared to vinclozolin lineage F3 generation germ cells from: (A) E13 and (B) E16. Gene node shape code: oval and circle – protein; diamond – ligand; circle/oval on tripod platform – transcription factor; ice cream cone – receptor. Grey connecters represent general regulation, blue – expression regulation, purple – binding, green – promoter binding. Network was derived using Pathway Studio™ software.

Analysis of the locations of the germ cell DMR (Table 2) with the differentially expressed genes (Tables S1 and S2) demonstrated no obvious correlation in either the E13 or E16 germ cell data sets. Therefore, the DMR found in specific gene promoters do not appear to regulate the adjacent genes expression at these stages of germ cell development. As previously observed (32), the presence of a DMR (epimutation) in a promoter does not generally correlate to altered expression of the adjacent gene. An alternate consideration is that indirect interactions between the DMR and gene expression may exist. The approach employed datasets from previous literature describing gene binding and functional relationships to identify potential indirect correlations between the germ cell DMR and differentially regulated gene sets, Figure 6. A number of DMR were found to be indirectly correlated with the germ cell differentially expressed genes at both the E13 and E16 stages of development. Therefore, minimal direct regulation of gene expression was observed in the DMR associated genes, but a number of potential indirect interactions were identified.

## Discussion

Environmentally induced epigenetic transgenerational inheritance requires germline transmission of altered epigenetic programming between generations in the absence of direct environmental exposures [2], [52]. A variety of environmental exposures, including toxicants [7] and nutrition [15], [16], can promote transgenerational phenotypes [2], [36]. The initial observations with vinclozolin indicated the transgenerational disease phenotype is transmitted through the male germline (sperm), but not through the female germ line (egg) [1]. The majority of subsequent studies have focused on paternal (sperm) transmission [7], [33]. Preliminary observations demonstrate DDT induces transgenerational phenotype (e.g. obesity) through the female germ line. Therefore, environmentally induced epigenetic transgenerational inheritance can involve either the male and/or female germ cells.

The critical window of exposure for induction of transgenerational epimutations in the mammal is during the later stages of primordial germ cell migration and colonization of the fetal gonad and during the initial stages of gonadal sex determination [1], [3], [29]. The PGCs undergo an erasure of DNA methylation prior to gonadal sex determination and then subsequent re-methylation in a sex-specific manner [4], [5], [37], [38], [39]. The hypothesis tested is that the exposure alters this epigenetic programming of the germline to develop new epimutations in the form of differential DNA methylation regions (DMR) that then, in a permanent imprinted-like manner, are transmitted to subsequent generations to promote the epigenetic transgenerational inheritance of disease and phenotypic variation [2], [36]. In the rat, the E13 stage of development involves the final stage of PGC development and initiation of gonadal sex determination when male germ cells transition to prospermatogonial cell differentiation. The exposure period during E8–E14 predominantly impacts PGC development. In contrast, at E16 the male germ cells are at the type T1 prospermatogonia stage which occurs following testis cord formation (42). The current study was designed to examine the transgenerational transcriptome and epigenome alterations at these two stages of germ cell development. The abnormal germ cell programming observed in F3 generation vinclozolin lineage descendants provides evidence that the original exposure to vinclozolin induces an altered germ cell epigenome that is transmitted transgenerationally.

Observations demonstrated 592 differentially expressed genes in vinclozolin lineage germ cells at E13, but only 148 differentially expressed genes at E16. PGCs at E13 have entered a unique “epigenetic ground state” represented by maximum erasure of DNA methylation [53], [54], whereas type T1 prospermatogonia at E16 are at a stage when global remethylation of the germline genome has initiated [37]. Therefore, a greater degree of DNA methylation erasure in germ cells at E13 relative to E16 may be related to the greater number of dysregulated genes detected at these stages of development. Interestingly, there was negligible overlap between the sets of dysregulated genes at these two stages, with only 25 genes in common, Figure 1 and Tables S1 and S2. Therefore, the E13 and E16 germ cells had predominately distinct transgenerational transcriptomes. Although there was negligible overlap in the transgenerational transcriptomes between the E13 and E16 stages, the majority of the functional gene categories impacted were similar, Figure 1. Therefore, the vinclozolin induced transgenerational germline transcriptomes appear to impact similar cellular processes in the E13 and E16 germ cells even though the specific gene sets affected are distinct.

A pathway analysis demonstrated the E13 gene list had over 20 different pathways altered while the vinclozolin lineage germ cells at E16 had only one pathway disrupted. The E13 germ cell differentially expressed genes influenced a variety of cellular pathways and processes which were found to be distinct from the E16 germ cells. One pathway that had 64 genes differentially expressed in vinclozolin lineage E13 germ cells was the olfactory transduction pathway, Figure 2. All the dysregulated genes encode olfactory receptors that have been shown to be under epigenetic regulation of the gene family [49], [50]. Olfactory receptors have been shown to be susceptible to transgenerational alterations [18], [34], and have also been shown to be involved in germ cell function and reproduction [55]. Future studies will need to examine the potential functional impact of the altered olfactory receptor family. Interestingly, a previous study demonstrated that sexual selection mate preference behavior was altered in the transgenerational F3 generation vinclozolin lineage animals [31], and it was speculated that this could in part be due to altered olfaction [31]. The current study supports the potential that altered regulation of the olfactory receptor gene family may contribute to the behavior modifications in vinclozolin lineage animals observed.

The final investigation of the germ cells transcriptome involved a gene network analysis. The E13 PGC and E16 prospermatogonia germ cell differentially expressed gene sets were used to create networks of genes based on interactions among genes and proteins reported in previous literature. A relatively large gene network was identified in differentially expressed E13 PGC genes, while at E16 the genes disrupted in germ cells formed a much smaller network. Future studies are now required to assess the functional importance of these transgenerational differentially expressed gene networks. Observations demonstrate a transgenerational effect on PGC and prospermatogonia germ cell transcriptomes.

The transgenerational PGC and prospermatogonia epigenomes were also assessed in F3 generation control versus vinclozolin lineage E13 and E16 animals. The germ cells from each developmental period showed predominantly unique, non-overlapping, differential DNA methylation regions (DMR). The analysis of DMR based on averages of three different experiments generated larger sets of DMR for each period, but still showed minimal overlap between E13 and E16 germ cells, Figure 4. Those DMR that were reproducible for all experiments were termed intersection DMR. Intersection DMR were almost entirely unique for the E13 and E16 germ cells with only one overlap which was found in the *Pigb* gene promoter. The prodigiosin biosynthetic gene cluster family member *Pigb* encodes phosphatidylinositol glycan class B. This protein has a variety of functions in a number of species [56], [57] from conferring mercury and copper resistance [58], [59] to facilitating signaling in cells [60], [61]. Therefore, the epigenetic alterations observed in vinclozolin lineage E13 PGCs were distinct from those in E16 prospermatogonia. Observations suggest an ongoing cascade of epigenetic alterations as germ cells develop during this period. Interestingly, the DMR identified in F3 generation vinclozolin lineage fetal germ cells showed similar genomic features as those previously described for F3 generation vinclozolin lineage sperm [6]. These include low CpG density and the presence of an adenosine rich DNA sequence motif, Figure 5. The number of CpG/100 bp was found to average 3.1 CpG/100 bp, such that these regions are CpG deserts, as previously described [6], [7]. Since C to T conversions are the most common base pair mutation in mammals, evolutionarily deserts of CpG develop in the mammalian genome [62]. The persistence of regions retaining clusters of CpGs suggests potential regulatory roles for these sites. In addition to low CpG density, many of the DMR shared a DNA sequence motif similar to the environmentally induced DNA methylation sequence motif1 (EDM1) previously identified in F3 generation vinclozolin lineage sperm [6]. Therefore, there appear to be specific genomic features that renders these sites susceptible to become transgenerationally programmed.

The current study demonstrates that ancestral exposure of a gestating female during fetal gonadal sex determination to the agricultural fungicide vinclozolin promotes a transgenerational alteration in germ cell epigenetic programming in the F3 generation (great grandchildren). The E13 PGC at the onset of fetal gonadal sex determination and the E16 prospermatogonia both showed transgenerational alterations in both their transcriptomes and epigenomes (DNA methylation), but these alterations were largely distinct between the two developmental stages. Therefore, the altered germ cell programming appears to involve a cascade of transcriptional and epigenetic events that promote germ cell mediated transgenerational inheritance [2], [35]. In addition to the DMR being distinct in vinclozolin lineage germ cells at E13 and E16, these DMR were both distinct from those previously identified in F3 generation vinclozolin lineage sperm [6]. Observations demonstrate a specific DMR is not programmed in the PGC and then the same DMR transmitted to the sperm, but instead a cascade of epigenetic and transcriptional events throughout germ cell development and spermatogenesis likely leads to the mature sperm DMR transmitting the epigenetic transgenerational phenotype. The current study used an MeDIP-Chip genome wide promoter analysis and did not investigate the entire genome. Therefore, DMR outside of promoter regions may show more similarity between the PGC and the sperm DMR, but this remains to be investigated. Future studies will require genome wide analysis to identify the cascade of epigenetic and transcriptional events at various germ cell developmental stages to correlate with the DMR in the mature germ cells.

Previous studies have demonstrated transgenerational male infertility and altered sperm motility is observed in the vinclozolin F3 generation males [1], [29]. Alterations in sperm epigenomes have also been linked to male infertility and other disease [63], [64], [65]. Results of the current study indicate the molecular basis of this transgenerational disease and male infertility is directly linked to the altered epigenetic programming of the PGC and subsequent germ cells investigated.

A comparison between the germ cell DMR and the differentially expressed genes indicated no significant overlap. This suggests minimal direct promoter regulation through a DMR for an adjacent gene at these stages of development. In contrast, some potential indirect gene associations were identified for both the E13 and E16 developmental stages between the DMR and differentially expressed genes, Figure 6. Although negligible direct promoter associations were observed, previously we have identified an epigenetic control region that may allow a DMR to distally regulate gene expression over a significant distance [34]. An “epigenetic control region” containing a DMR in proximity to a long non-coding RNA (lncRNA) may regulate gene expression for over a 2–5 megabase region [34]. This epigenetic control of gene expression provides an alternate mechanism for the DMR identified to regulate distally the PGC and prospermatogonia differentially expressed genes observed. Future studies will need to correlate the cascade of epigenetic and transcriptional events in the developing germ cells to these types of epigenetic control regions.

The combined observations demonstrate ancestral exposure of a gestating female during fetal gonadal sex determination can promote transgenerational alterations of normal germline epigenetic and transcriptional programming that leads to the epigenetic transgenerational inheritance of disease and phenotypic variation. Observations support a role for disrupted germline epigenetic programming in the etiology of the epigenetic transgenerational inheritance phenomenon. Results suggest a cascade of epigenetic and transcriptional events during germ cell development is needed to obtain the mature germline epigenome involved in transgenerational transmission of the epigenetic inheritance.

## Methods

### Animals and Exposures

Hsd Sprague Dawley®™SD®™ female and male rats of an outbred strain (Harlan) were maintained in ventilated (up to 50 air exchanges/hour) isolator cages (with dimensions of 10 ¾” W x 19 ¼“ D x 10 ¾” H, 143 square inch floor space, fitted in Micro-vent 36-cage rat racks; Allentown Inc., Allentown, NJ) containing Aspen Sani chips (pinewood shavings from Harlan) as bedding, on a 14 h light/10 h dark regimen, at a temperature of 70 F and humidity of 25% to 35%. Rats were fed ad libitum with standard rat diet (8640 Teklad 22/5 Rodent Diet; Harlan) and *ad libitum* tap water for drinking. At pro-estrus as determined by daily vaginal smears, the female rats (90 days of age) were pair-mated with male rats (120 days). On the next day, the pairs were separated and vaginal smears were examined microscopically. If sperm were detected (day 0) the rats were tentatively considered pregnant. Monitoring of vaginal smears was continued for diestrus status in these rats until day 7. Pregnant rats for the treatment group were given daily intraperitoneal injections of vinclozolin (100 mg/kg BW/d; Chem Service, West Chester, PA) and an equal volume of sesame oil (Sigma) on days E8 through E14 of gestation; Vinclozolin was dissolved in DMSO (Sigma). Pregnant rats for the control group were given daily intraperitoneal injections of DMSO (100 ul/kg BW/d) and an equal volume of sesame oil (Sigma) on days E8 through E14 of gestation [66]. The pregnant female rats treated with vinclozolin were designated as the F0 generation. All experimental protocols for the procedures with rats were pre-approved by the Washington State University Institutional Animal Care and Use Committee (IACUC approval # 02568-030).

### Breeding F1, F2, and F3 Generations

The offspring of the F0 generation were the F1 generation. The F1 generation offspring were bred to other F1 animals of the same treatment group to generate an F2 generation, and then F2 generation animals were bred similarly to generate the F3 generation animals. No sibling or cousin breeding was performed so as to avoid inbreeding. Note that only the original F0 generation pregnant females were injected with vinclozolin or vehicle.

### Fetal Gonadal Germ Cell Preparation

Harlan Sprague-Dawley rats (Harlan Inc, Indianapolis IN) were used for all experiments. The rats were kept in a temperature controlled environment and given food and water *ad libitum*. Estrous cycles of female rats were monitored by cellular morphology from vaginal smears. Rats in early estrus were paired with males overnight and mating confirmed by sperm-positive smears, denoted day 0 of pregnancy. Pregnant rats were euthanized at embryonic day 13 (E13) or 16 (E16) of gestation, and fetal gonads were collected for germ cell preparations. At E13, sex was determined by PCR on genomic DNA isolated from embryo tails using primers specific for the *Sry* gene as previously described [43]. At E16, sex was determined on the basis of gonadal morphology. Germ cells were isolated exclusively from males.

Purified populations of male PGCs (at E13) or type T1 prospermatogonia (at E16) were prepared using a mini StaPut gradient method as previously described [67], [68]. Briefly, fetal testes were pooled and dissociated by incubation in 0.25% trypsin-EDTA (Sigma) with vigorous pipetting using a 1000 microliter pipette tip, and the resulting cell solution was filtered through 100 micron nylon mesh to yield a single cell suspension. This cell suspension was then loaded onto a 50 ml 2–4% bovine serum albumen (BSA) gradient prepared in KREBS buffer, and the cells were allowed to sediment at unit gravity at 4°C for two hours as described [67], [68]. The gradient was then fractionated and aliquots of the fractions were examined under phase optics to identify those enriched for the appropriate PGC or prospermatogonial cell types on the basis of morphological characteristics. The enriched fractions were pooled to yield the final sample which was ≥85% pure for the desired male germ cell type in each case.

### RNA Extraction and Microarray Transcriptome Analysis

Messenger RNA was isolated using the Trizol^TM^ (Invitrogen) method per the manufacturer’s protocol. Messenger RNA was independently extracted from 3 pools of germ cells (i.e. 3 biological replicates) per treatment. The mRNA processing and hybridizations were performed at the Genomics Core Laboratory, Center for Reproductive Biology, Washington State University, Pullman, WA using standard Affymetrix reagents and protocols. Briefly, mRNA was reverse transcribed into cDNA with random primers, then cRNA was transcribed from the cDNA, and from that, single-stranded sense DNA was synthesized which was fragmented and labeled with biotin. Biotin-labeled, fragmented ssDNA was then hybridized to the Rat Gene 1.0 ST microarrays containing more than 27,000 transcripts (Affymetrix, Santa Clara, CA, USA). Hybridized chips were scanned on an Affymetrix Scanner 3000. CEL files containing raw data were then pre-processed and analyzed with Partek Genomic Suite 6.5 beta software (Partek Incorporated, St. Louis, MO) using an RMA and GC-content adjusted algorithm (Figure S1). The signals from an average of 28 different probes for each transcript were compared to give a single value. Two-way ANOVA was performed between the germ cell transcriptomes from F3 generation vinclozolin and control lineage cells. One factor of variation was treatment and the other was batch effect. Corrections were made for cell preparation date batch effect by the Partek software according to the Methods of Moments [69]. The selection of the differentially expressed genes was based on the expression change between vinclozolin and control lineage germ cells limited to p-values <0.05, expression fold change >1.2, and the mean difference between vinclozolin and control un-logged signals >10. CEL files from this study have been deposited with the NCBI gene expression and hybridization array data repository (GEO, http://www.ncbi.nlm.nih.gov/geo, GEO # GSE43559) and can also be accessed through www.skinner.wsu.edu. For gene annotation, the Affymetrix annotation file RaGene1_0stv1.na31.rn4.transcript.csv was used unless otherwise specified.

### Pathway and Gene Network Analysis

Known functional relationships among the F3 generation differentially expressed genes were identified using the KEGG pathways from the University of Kyoto (Japan) Encyclopedia for Genes and Genome website (http://www.genome.jp/_eg/) and Pathway Express (http://vortex.cs.wayne.edu) [63]. Functional relationships among the F3 generation differentially expressed genes and genes with changes in DNA methylation were also interrogated using Pathway Studio software (Ariadne, Genomics Inc. Rockville MD), using an unbiased, automated survey of published scientific literature (Global Literature Analysis). This analysis identifies functional relations among genes, such as direct binding, up-regulation or down-regulation and also builds sub-networks of genes and cellular processes based on their inter-connections.

### DNA Extraction and Methylated DNA Immunoprecipitation (MeDIP)

DNA was isolated using the Trizol^TM^ (Invitrogen) method per the manufacturer’s protocol, from the same germ cell Trizol^TM^ preparations that were used for RNA isolations. Therefore, three independent DNA Trizol^TM^ fractions from germ cells per group were used to obtain three different biological replicates of DNA samples from each of the two treatment groups. Each of these DNA samples were then used for methylated DNA immunoprecipitation (MeDIP). MeDIP was performed as follows: 1 µg of genomic DNA was subjected to a series of three 20 pulse sonications at 20% amplitude. The appropriate fragment size (200–1000 bp) was verified using 2% agarose gels. The sonicated genomic DNA was resuspended in 350 ul TE and denaturated for 10 min at 95°C and then immediately placed on ice for 5 min; 100 ul of 5X IP buffer (50 mM Na-phosphate pH7, 700 mM NaCl, 0.25% Triton X-100) was added to the sonicated and denatured DNA. An overnight incubation of the DNA was performed with 5 ug of anti-5-methylCytidine monoclonal antibody from Diagenode S.A (Denville, NJ) at 4°C on a rotating platform. Protein A/G beads from Santa Cruz (Santa Cruz, CA) were prewashed with PBS-BSA 0.1% and resuspended in 40 ul 1X IP buffer. Beads were then added to the DNA-antibody complex and incubated 2 h at 4°C on a rotating platform. Beads bound to DNA-antibody complex were washed 3 times with 1 ml 1X IP buffer; washes included incubation for 5 min at 4°C on a rotating platform and then centrifugation at 6000 rpm for 2 min. Beads-DNA-antibody complexes were then resuspended in 250 ul digestion buffer (50 mM Tris HCl pH 8, 10 mM EDTA, 0.5% SDS) and 3.5 ul of proteinase K (20 mg/ml) was added to each sample and then incubated overnight at 55°C on a rotating platform. DNA purification was performed first with phenol and then with chloroform:isoamyl alcohol. Two washes were then performed with 70% ethanol, 1 M NaCl and glycogen. MeDIP selected DNA was then resuspended in 30 ul TE buffer. Whole-genome amplification was then performed with the WGA2 kit (Sigma-Aldrich) on each MeDIP sample to be used in the microarray comparative hybridization analysis.

### Tilling Array and MeDIP-Chip Bioinformatic and Statistical Analyses

Roche Nimblegen’s Rat DNA Methylation 3×720K CpG Island Plus RefSeq Promoter Array was used, which contains three identical sub-arrays, with 713,670 probes per sub-array, scanning a total of 15,287 promoters (3,880 bp upstream and 970 bp downstream from each transcription start site). Probe sizes range from 50–75 nucleotides in length with a median inter-probe spacing of 100 bp. Three different comparative (amplified MeDIP vs. amplified MeDIP) hybridization experiments included in three sub-arrays were performed by Nimblegen. Each comparative hybridization experiment contained one biological replicate of a germ cell whole genome amplified-MeDIP-DNA sample from each lineage treatment (control or vinclozolin lineages). Vinclozolin lineage MeDIP DNA samples were labeled with Cy3 and control lineage MeDIP DNA samples were labeled with Cy5. For each comparative hybridization experiment, raw data from both the Cy3 and Cy5 channels were imported into R (R Development Core Team (2010), R: A language for statistical computing, R Foundation for Statistical Computing, Vienna, Austria. ISBN 3-900051-07-0, URL http://www.R-project.org), checked for quality and converted to MA values (M =  Cy5-Cy3; A =  (Cy5+Cy3)/2). The following normalization procedure was conducted within each array. Probes were separated into groups by GC content and each group was separately normalized between Cy3 and Cy5 using the loess normalization procedure. Normalization curves were generated specific to each GC group. The arrays were then normalized across arrays using the A quantile normalization procedure. Following normalization, each probe within each array was normalized and M values were replaced with the median value of all probe normalized M values across all arrays within a 600 bp window. If the number of probes present in the window was less than 3, no value was assigned to that probe. Each probe’s A values were likewise normalized using the same procedure. Following normalization, each probe’s M value represented the median intensity difference between vinclozolin lineage and control lineage samples within a 600 bp window. Significance was assigned to probe differences between vinclozolin lineage and control lineage samples by calculating the median value of the intensity differences as compared to a normal distribution scaled to the experimental mean and standard deviation of the normalized M. A Z-score and P-value were computed for each probe from that distribution. The statistical analysis was performed in pairs of comparative IP hybridizations between vinclozolin lineage (V) and control lineage I. V1-C1 and V2-C2 gave 715 sites; V1-C1 and V3-C3 gave 633 sites; V2-C2 and V3-C3 gave 807 sites (multiple sites exist within a specific DMR). In order to assure the reproducibility of the candidate DMR obtained, only the candidate DMR showing significant changes in all three of the paired comparisons were chosen as having a significant change in DNA methylation between the vinclozolin lineage and control lineage samples. This is a very stringent approach to select for differences, since it only considers those differences found in all paired analyses.

The DNA sequence motif analysis for the germ cell DMR identified used the Glam2 tool from MEME SUITE [70] as previously described [6].

## Supporting Information

Figure S1 **Sample histograms and box plots for germ cell RNA expression microarray probe signal intensity values after pre-processing with an RMA, GC-content adjusted algorithm.** Plots for F3 generation control and vinclozolin lineage germ cells from E13 and E16.
(PDF)

Table S1 **Genes differentially expressed in E13 F3 generation primordial germ cells.** The 25 genes that were also found among genes differentially expressed in F3 generation prospermatogonia at E16 are marked by bold font.
(PDF)

Table S2 **Genes differentially expressed in E16 F3 generation germ cells.** The 25 genes that were also found among genes differentially expressed in F3 generation PGCs at E13 are marked by bold font.
(PDF)
