# Correction: Environmentally Induced Transgenerational Epigenetic Reprogramming of Primordial Germ Cells and the Subsequent Germ Line

**DOI:** 10.1371/annotation/7683bb48-85db-4c7e-87c0-304a7d53a587

**Published:** 2013-07-30

**Authors:** Michael K. Skinner, Carlos Guerrero-Bosagna M. Haque, Eric Nilsson, Ramji Bhandari, John R. McCarrey

The names of the second and third authors were incorrectly joined in the byline and citation. The correct names of the second and third authors are Carlos Guerrero-Bosagna and M. Haque, respectively. 

The correct citation is: Skinner MK, Guererro-Bosagna C, Haque M, Nilsson E, Bhandari R, McCarrey JR (2013) Environmentally Induced Transgenerational Epigenetic Reprogramming of Primordial Germ Cells and the Subsequent Germ Line. PLoS ONE 8(7): e66318. doi:10.1371/journal.pone.0066318. 

Both authors are affiliated with institution 1, Center for Reproductive Biology, School of Biological Sciences, Washington State University, Pullman, Washington, United States of America. 

