# Supplementary figures and images for: Environmentally Induced Transgenerational Epigenetic Reprogramming of Primordial Germ Cells and the Subsequent Germ Line
Source: PLoS One. 2013 Jul 15;8(7):e66318. doi: 10.1371/journal.pone.0066318 (PMC3712023; doi:10.1371/journal.pone.0066318)

Supplemental Figure S1

Microarray Hybridization Profiles

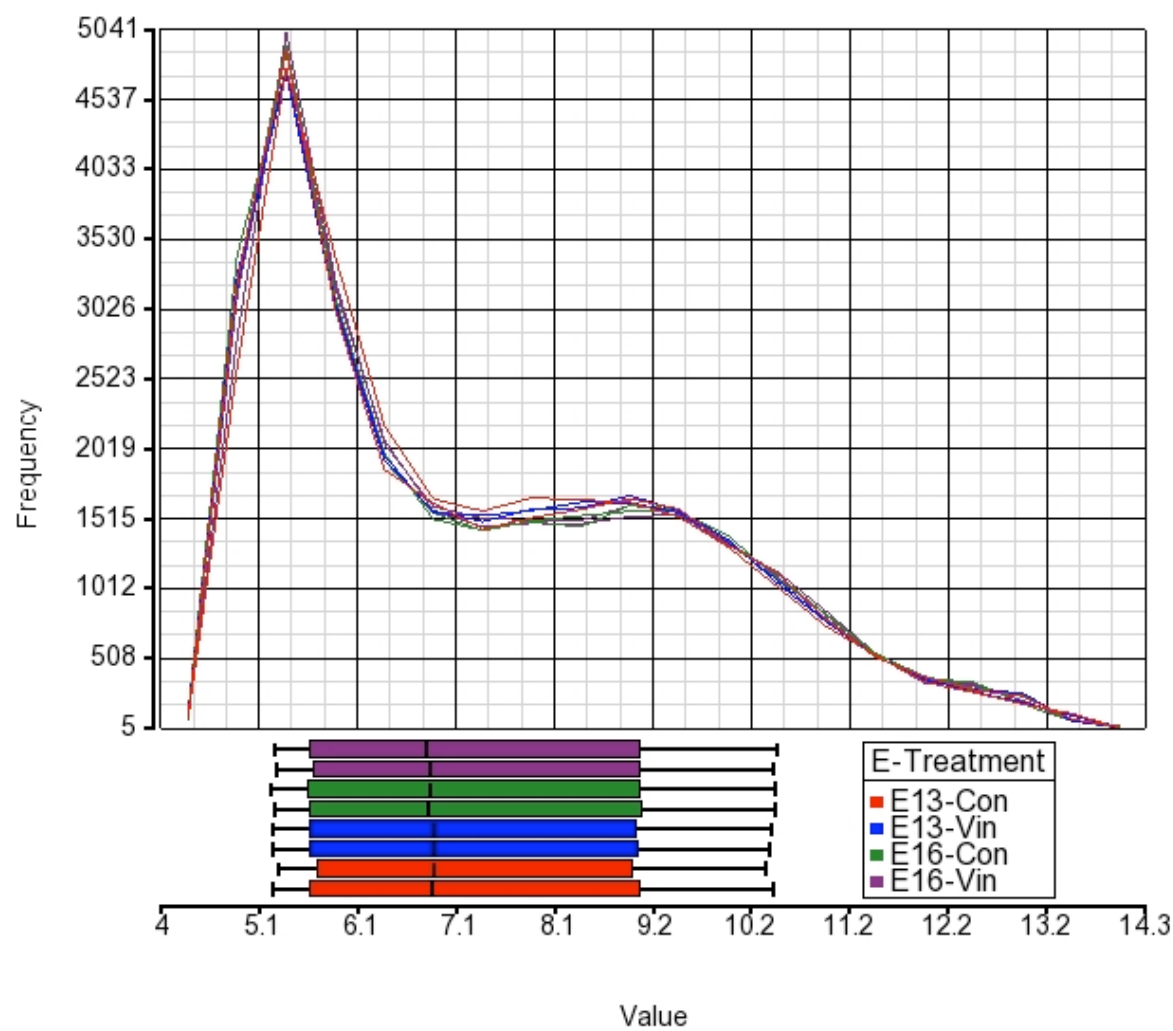

Supplement: Figure S1 — Sample histograms and box plots for germ cell RNA expression microarray probe signal intensity values after pre-processing with an RMA, GC-content adjusted algorithm. Plots for F3 generation control and vinclozolin lineage germ cells from E13 and E16. (PDF) [file pone.0066318.s001.pdf]
