# Supplementary material for: Environmentally Induced Transgenerational Epigenetic Reprogramming of Primordial Germ Cells and the Subsequent Germ Line
Source: PLoS One. 2013 Jul 15;8(7):e66318. doi: 10.1371/journal.pone.0066318 (PMC3712023; doi:10.1371/journal.pone.0066318)
Supplement: Table S1 — Genes differentially expressed in E13 F3 generation primordial germ cells. The 25 genes that were also found among genes differentially expressed in F3 generation prospermatogonia at E16 are marked by bold font. (PDF) [file pone.0066318.s002.pdf]

Supplemental Table S1

Genes Differentially Expressed in E13 F3 Generation Primordial Germ Cells

**GENE FUNCTIONAL GROUP/** Gene Symbol	**RefSeq/GenBank/ Ensemble Number**	**Affymetrix Probset ID**	**Vin/Con**	**Mean Diff.**	**Gene Title**
**Apoptosis**					
Aven	NM_001107757	10838512	1.23	79	apoptosis, caspase activation inhibitor
Moap1	NM_001013101	10891861	1.28	24	modulator of apoptosis 1
RGD1559610	BC168964	10879679	1.22	125	similar to CGI-94 protein
Tnfsf14	NM_001191803	10931712	0.81	-10	tumor necrosis factor (ligand) superfamily, member 14
**Cell Cycle**					
Ccne2	NM_001108656	10867614	1.36	327	cyclin E2
Cdkn2c	NM_131902	10878705	1.69	81	cyclin-dependent kinase inhibitor 2C (p18, inhibits CDK4)
Cinp	NM_001106758	10892212	1.29	63	cyclin-dependent kinase 2-interacting protein
**Cytoskeleton-ECM**					
Mxra8	NM_001007002	10882224	0.83	-11	matrix-remodelling associated 8
Actn2	NM_001170325	10795736	0.83	-10	actinin alpha 2
Actr10	NM_001009602	10885031	1.24	331	actin-related protein 10 homolog (S. cerevisiae)
Bfsp1	NM_031555	10850352	1.22	19	beaded filament structural protein 1
Cdh19	NM_001009448	10767016	2.74	54	cadherin 19, type 2
Cno	NM_001100766	10773331	1.33	19	cappuccino homolog (mouse)
Cotl1	NM_001108452	10811452	1.25	127	coactosin-like 1 (Dictyostelium)
Dnah9	D26500	10743677	0.78	-17	dynein, axonemal, heavy polypeptide 9
Erc2	NM_170787	10786382	1.28	15	ELKS/RAB6-interacting/CAST family member 2
Erlec1	NM_001106023	10778897	1.27	277	endoplasmic reticulum lectin 1
Gpm6a	NM_178105	10791552	1.42	86	glycoprotein m6a
Krt15	NM_001004022	10747252	0.79	-13	keratin 15
Krt40	ENSRNOT00000045189	10747139	0.78	-67	keratin 40
Krt83	NM_001101675	10907357	0.80	-19	keratin 83
Krtap31-1	NM_001109409	10738168	0.78	-133	keratin associated protein 31-1
Krtap4-5	ENSRNOT00000068237	10747166	0.70	-28	keratin associated protein 4-5
Krtap5-5	ENSRNOT00000047885	10726935	0.71	-171	keratin associated protein 5-5
Lgals2	NM_133599	10905356	1.45	32	lectin, galactoside-binding, soluble, 2
Lgals8	NM_053862	10795724	1.29	79	lectin, galactoside-binding, soluble, 8
LOC292516	NM_001013893	10718462	0.75	-12	similar to actin-like 7-alpha-like protein
Myo1d	NM_012983	10745509	1.37	85	myosin ID
**Pcdh15**	**ENSRNOT00000066040**	**10832646**	**0.56**	**-57**	**protocadherin 15**
Pcdh7	NM_001004087	10776978	0.72	-117	protocadherin 7
RGD1566146	ENSRNOT00000047104	10747185	0.74	-103	similar to keratin associated protein 4-10
Twf1	NM_001008521	10906533	1.26	203	twinfilin, actin-binding protein, homolog 1 (Drosophila)
**Development**					
Brp44l	NM_133561	10718102	1.33	76	brain protein 44-like
Cisd2	NM_001191608	10826914	1.26	73	CDGSH iron sulfur domain 2
Cops3	NM_001004200	10743186	1.21	329	COP9 constitutive photomorphogenic homolog subunit

					3 (Arabidopsis)
Cyfip2	NM_001106996	10742254	1.39	75	cytoplasmic FMR1 interacting protein 2
Dynlt3	NM_001013228	10932366	1.24	70	dynein light chain Tctex-type 3
Fez2	NM_053600	10887831	1.35	46	fasciculation and elongation protein zeta 2 (zygin II)
Fkrp	NM_001025678	10719291	1.24	44	fukutin related protein
Gdap1l1	NM_001107798	10842001	1.36	36	ganglioside-induced differentiation-associated protein 1-like 1
Htatip2	NM_001106263	10707230	1.47	49	HIV-1 tat interactive protein 2, homolog (human)
Lce1d	ENSRNOT00000012464	10817123	0.71	-24	late cornified envelope 1D
Lcn12	NM_001128138	10843468	0.83	-26	lipocalin 12
**LOC171573**	**NM_138537**	**10909012**	**0.75**	**-22**	**spleen protein 1 precursor**
LOC685769	ENSRNOT00000042069	10772840	0.79	-50	similar to Discs large homolog 5 (Placenta and prostate DLG) (Discs large protein P-dlg)
Lrrc56	NM_001024902	10712224	0.77	-35	leucine rich repeat containing 56
Lrrc57	NM_001012354	10839043	1.20	64	leucine rich repeat containing 57
Mlc1	NM_001108105	10906079	0.81	-11	megalencephalic leukoencephalopathy with subcortical cysts 1 homolog (human)
**Mospd1**	**NM_001014107**	**10939816**	**1.31**	**149**	**motile sperm domain containing 1 (chrX)**
Mospd1	NM_001014107	10778399	1.31	160	motile sperm domain containing 1 (chr14)
Myog	NM_017115	10764092	0.75	-12	myogenin
Osr1	NM_001106716	10883586	0.72	-16	odd-skipped related 1 (Drosophila)
Pno1	NM_199083	10778568	1.47	379	partner of NOB1 homolog (S. cerevisiae)
RGD1560580	ENSRNOT00000048690	10758104	0.52	-77	similar to spermiogenesis specific transcript on the Y 2 (chr12)
RGD1560580	ENSRNOT00000048690	10791327	0.54	-50	similar to spermiogenesis specific transcript on the Y 2 (chr14)
Sema4b	NM_001170462	10708154	1.21	92	sema domain, immunoglobulin domain (Ig), transmembrane domain (TM) and short cytoplasmic domain, (semaphorin) 4B
Sprr1al	ENSRNOT00000012174	10824732	0.66	-25	small proline-rich protein 1A-like
Sprr1b	ENSRNOT00000040171	10824726	0.82	-18	small proline-rich protein 1B (cornifin)
Surf1	NM_172068	10843881	1.41	81	surfeit 1
Suv420h2	NM_001107475	10718873	0.80	-76	suppressor of variegation 4-20 homolog 2 (Drosophila)
Tex101	NM_139037	10719674	1.63	439	testis expressed 101
Trpc6	NM_053559	10907962	1.63	55	transient receptor potential cation channel, subfamily C, member 6
Vcsa2	NM_198729	10776116	0.77	-12	variable coding sequence A2
Wrb	NM_199373	10750473	1.20	150	tryptophan rich basic protein
**DNA Repair**					
Rad52	NM_001106617	10858260	0.83	-63	RAD52 homolog (S. cerevisiae)
**Electron Transport**					
Cyp2c22	NM_138512	10730046	0.74	-10	cytochrome P450, family 2, subfamily c, polypeptide 22
LOC679879	ENSRNOT00000061522	10786123	0.59	-65	similar to Spetex-2F protein
Tmem126a	NM_001011557	10723639	1.27	74	transmembrane protein 126A
Txndc16	ENSRNOT00000008372	10782804	1.53	100	thioredoxin domain containing 16
Txnl1	NM_080887	10804856	1.20	494	thioredoxin-like 1
**Epigenetics**					
Coq3	NM_019187	10875756	1.27	207	coenzyme Q3 homolog, methyltransferase (S. cerevisiae)
**Golgi Apparatus**					
Gal3st2	ENSRNOT00000038687	10925678	0.77	-17	galactose-3-O-sulfotransferase 2

**Growth Factors, Cytokines, Hormones**					
Cmklr1	NM_022218	10759198	1.48	50	chemokine-like receptor 1
Hdgfl1	NM_133549	10795072	0.77	-43	hepatoma derived growth factor-like 1
Pyy	NM_001034080	10747735	0.77	-13	peptide YY (mapped)
Retn	NM_144741	10759611	0.78	-14	resistin
Sept7	NM_022616	10908674	1.23	295	septin 7
Pthlh	NM_012636	10866970	0.83	-44	parathyroid hormone-like hormone
Gcg	NM_012707	10845676	0.20	-80	glucagon
**Immune Response**					
Ahsg	NM_012898	10755157	2.13	78	alpha-2-HS-glycoprotein
Ambp	NM_012901	10877448	1.51	21	alpha-1-microglobulin/bikunin precursor
Cd80	NM_012926	10754176	1.36	25	Cd80 molecule
Cfd	NM_001077642	10900735	1.28	12	complement factor D (adipsin)
Ifna4	NM_001106667	10877954	0.78	-15	interferon, alpha 4
IgG-2a	BC088240	10892506	0.70	-114	gamma-2a immunoglobulin heavy chain
Igh-6	BC092586	10892653	0.57	-35	immunoglobulin heavy chain 6
LOC682821	ENSRNOT00000031103	10862947	0.47	-23	pre-B lymphocyte 3-like
Ly6g6c	NM_001001969	10831236	0.72	-41	lymphocyte antigen 6 complex, locus G6C
Ly75	ENSRNOT00000010683	10845517	1.20	16	lymphocyte antigen 75
Magea10	ENSRNOT00000052261	10935804	0.79	-11	melanoma antigen family A, 10
RGD1564284	ENSRNOT00000044584	10892509	0.64	-36	similar to immunoglobulin 4G6 heavy chain variable region
RGD1564392	ENSRNOT00000067463	10887662	0.73	-107	similar to immunoglobulin heavy chain variable region
Vpreb2	NM_001134788	10755629	0.80	-54	pre-B lymphocyte gene 2
Xlr3a	ENSRNOT00000040782	10940120	1.39	56	X-linked lymphocyte-regulated 3A
**Metabolism & Transport**					
Abhd10	NM_001123352	10751018	0.82	-151	abhydrolase domain containing 10
Agpat5	NM_001134744	10792592	1.30	706	1-acylglycerol-3-phosphate O-acyltransferase 5 (lysophosphatidic acid acyltransferase, epsilon)
Aldh9a1	NM_022273	10765399	1.21	232	aldehyde dehydrogenase 9 family, member A1
Aldob	NM_012496	10876719	1.61	63	aldolase B, fructose-bisphosphate
Aqp2	NM_012909	10899145	0.69	-21	aquaporin 2 (collecting duct)
Aqp6	NM_022181	10899139	0.81	-10	aquaporin 6, kidney specific
Atp1b3	NM_012913	10910191	1.27	533	ATPase, Na+/K+ transporting, beta 3 polypeptide
B3galt6	NM_001106699	10882246	1.21	19	UDP-Gal:betaGal beta 1,3-galactosyltransferase, polypeptide 6
Catsperg1	NM_001170340	10720437	0.65	-84	cation channel, sperm-associated, gamma 1
Clcn1	NM_013147	10855035	0.83	-10	chloride channel 1
Cmc1	NM_001135259	10920797	1.70	89	COX assembly mitochondrial protein homolog (S. cerevisiae)
Echdc1	NM_001007734	10717413	1.22	55	enoyl Coenzyme A hydratase domain containing 1
Eno3	NM_012949	10735331	0.76	-41	enolase 3, beta, muscle
Hdc	NM_017016	10849522	1.76	34	histidine decarboxylase
**Hmox1**	**NM_012580**	**10806122**	**1.21**	**34**	**heme oxygenase (decycling) 1**
Kcnj10	NM_031602	10765740	0.83	-14	potassium inwardly-rectifying channel, subfamily J, member 10
Kcnk18	NM_001003820	10716430	0.71	-27	potassium channel, subfamily K, member 18
Klk1l	NM_012593	10706488	0.63	-33	kallikrein 1-like peptidase
Klk5l	ENSRNOT00000056456	10706474	0.80	-10	kallikrein 5-like
Lactb2	NM_001024247	10867165	1.40	153	lactamase, beta 2
Lpcat1	NM_001100735	10717571	1.22	170	lysophosphatidylcholine acyltransferase 1

Lpl	NM_012598	10791250	1.41	44	lipoprotein lipase
Lrp1b	NM_001107843	10845057	0.79	-13	low density lipoprotein-related protein 1B (deleted in tumors)
Moxd1	ENSRNOT00000061234	10717240	1.77	246	monooxygenase, DBH-like 1
Mthfd1l	BC166462	10702626	1.25	59	methylenetetrahydrofolate dehydrogenase (NADP+ dependent) 1-like
Mthfd2l	NM_001107211	10775885	1.20	13	methylenetetrahydrofolate dehydrogenase (NADP+ dependent) 2-like
Nt5dc3	NM_001134887	10894660	1.33	37	5'-nucleotidase domain containing 3
RGD1560203	ENSRNOT00000041781	10937824	0.64	-63	similar to ferritin heavy polypeptide-like 17
Slc25a10	NM_133418	10740159	1.23	113	solute carrier family 25 (mitochondrial carrier; dicarboxylate transporter), member 10
**Slc25a25**	**NM_145677**	**10844339**	**1.25**	**21**	**solute carrier family 25 (mitochondrial carrier, phosphate carrier), member 25**
Slc25a26	NM_001108638	10857412	0.79	-56	solute carrier family 25, member 26
Slc25a32	NM_001173334	10903482	1.20	88	solute carrier family 25, member 32
Slc35a2	NM_001127642	10936987	0.82	-33	solute carrier family 35, member A2
Slc6a2	NM_031343	10809507	0.72	-20	solute carrier family 6 (neurotransmitter transporter, noradrenalin), member 2
St3gal5	NM_031337	10856357	1.38	212	ST3 beta-galactoside alpha-2,3-sialyltransferase 5
Tmem161a	NM_001108874	10791058	0.82	-60	transmembrane protein 161A
Trappc2	NM_001024965	10937660	1.25	205	trafficking protein particle complex 2
Ugp2	NM_001024743	10778647	1.27	370	UDP-glucose pyrophosphorylase 2
Uxs1	NM_139336	10931543	1.25	316	UDP-glucuronate decarboxylase 1
**Proteolysis**					
Dcun1d2	ENSRNOT00000026370	10789470	1.27	26	DCN1, defective in cullin neddylation 1, domain containing 2 (S. cerevisiae)
LOC498793	BC100087	10796173	1.91	39	similar to inter-alpha-inhibitor H2 chain
LOC686393	ENSRNOT00000057510	10863662	0.81	-168	similar to Protease-associated domain-containing protein of 21 kDa precursor
Mcpt1l2	ENSRNOT00000038253	10780574	0.75	-17	mast cell protease 1-like 2
Mcpt1l3	ENSRNOT00000045948	10780588	0.74	-19	mast cell protease 1-like 3
Prss2	ENSRNOT00000029319	10854953	0.78	-15	protease, serine, 2
RGD1565317	ENSRNOT00000017230	10736332	1.32	123	similar to ubiquitin-like/S30 ribosomal fusion protein
Rlim	NM_001024892	10938841	1.27	319	ring finger protein, LIM domain interacting
Rnf103	NM_053438	10856282	1.29	173	ring finger protein 103
Slpil3	NM_001008873	10851577	0.78	-27	antileukoproteinase-like 3
Tmprss4	NM_001108998	10916963	0.83	-10	transmembrane protease, serine 4
Ube2v2	NM_183052	10752621	1.35	168	ubiquitin-conjugating enzyme E2 variant 2
Vps33b	NM_022286	10708188	1.27	102	vacuolar protein sorting 33 homolog B (yeast)
Vps53	NM_001105813	10744999	1.27	179	vacuolar protein sorting 53 homolog (S. cerevisiae)
**Receptors & Binding Proteins**					
Adipor2	NM_001037979	10865081	1.31	390	adiponectin receptor 2
Fgfbp1	NM_022603	10773146	0.76	-16	fibroblast growth factor binding protein 1
Hbxip	NM_001106462	10818257	1.28	123	hepatitis B virus x interacting protein
Itgb1bp2	NM_001108245	10934394	0.80	-17	integrin beta 1 binding protein 2
LOC100364003	ENSRNOT00000048946	10718391	0.66	-41	vomeronasal 2, receptor 42-like
Ltb4r	NM_021656	10780505	0.83	-12	leukotriene B4 receptor

Ly49s4	AY649838	10866180	0.66	-27	Ly49 stimulatory receptor 4
Olr1014	NM_001000699	10893397	0.64	-19	olfactory receptor 1014
Olr105	NM_001000146	10724274	0.73	-17	olfactory receptor 105
Olr1051	NM_001001364	10900077	0.57	-23	olfactory receptor 1051
Olr1059	NM_001001362	10900085	0.75	-15	olfactory receptor 1059
Olr1071	NM_001000063	10900099	0.66	-17	olfactory receptor 1071
Olr1093	NM_001001388	10901100	0.59	-27	olfactory receptor 1093
Olr1104	NM_001000906	10906846	0.80	-10	olfactory receptor 1104
Olr113	NM_001000151	10724282	0.73	-11	olfactory receptor 113
Olr1135	NM_001000878	10915265	0.67	-12	olfactory receptor 1135
Olr1137	NM_001001079	10915267	0.83	-11	olfactory receptor 1137
Olr114	NM_001000951	10724284	0.64	-17	olfactory receptor 114
Olr1143	NM_001001017	10908171	0.68	-15	olfactory receptor 1143
Olr1159	NM_001000871	10908191	0.70	-13	olfactory receptor 1159
Olr1186	NM_001000862	10908237	0.63	-22	olfactory receptor 1186
Olr1203	NM_001001082	10909117	0.72	-11	olfactory receptor 1203
Olr1234	NM_001000446	10909170	0.63	-18	olfactory receptor 1234
Olr1280	NM_001000517	10916319	0.77	-14	olfactory receptor 1280
Olr1329	NM_001000791	10909305	0.74	-9.97	olfactory receptor 1329
Olr1330	NM_001000476	10916410	0.74	-23	olfactory receptor 1330
Olr1355	NM_214823	10749816	0.82	-10	olfactory receptor 1355
Olr1364	NM_001000858	10740780	0.71	-41	olfactory receptor 1364
Olr1384	NM_001002291	10742363	0.74	-13	olfactory receptor gene Olr1384
Olr1385	NM_214833	10742365	0.68	-26	olfactory receptor 1385
Olr1387	NM_001000001	10733072	0.69	-31	olfactory receptor 1387
Olr139	NM_001000161	10709469	0.81	-22	olfactory receptor 139
Olr1392	NM_001001090	10733082	0.76	-12	olfactory receptor 1392
Olr14	NM_001000695	10723503	0.75	-10	olfactory receptor 14
Olr1425	NM_001000010	10742940	0.75	-14	olfactory receptor 1425
Olr1440	NM_001000017	10742958	0.78	-22	olfactory receptor 1440
Olr1450	NM_001000774	10733892	0.78	-14	olfactory receptor 1450
Olr1455	NM_001000771	10733902	0.71	-35	olfactory receptor 1455
Olr1471	NM_001000722	10735733	0.58	-73	olfactory receptor 1471
Olr1481	NM_001000527	10735735	0.73	-23	olfactory receptor 1481
Olr1509	NM_001000528	10744819	0.79	-12	olfactory receptor 1509
Olr1515	NM_001001098	10735765	0.67	-22	olfactory receptor 1515
Olr1531	NM_001001102	10750612	0.75	-14	olfactory receptor 1531
Olr1576	NM_001000499	10765813	0.76	-13	olfactory receptor 1576
Olr1585	NM_001001109	10765829	0.75	-14	olfactory receptor 1585
Olr1602	NM_001000909	10765910	0.73	-10	olfactory receptor 1602
Olr1611	ENSRNOT00000016721	10779768	0.64	-12	olfactory receptor 1611
Olr1612	NM_001000096	10783036	0.72	-15	olfactory receptor 1612
Olr1625	NM_001000971	10783044	0.67	-16	olfactory receptor 1625
Olr1697	NM_001001111	10830800	0.69	-18	olfactory receptor 1697
Olr1701	NM_001001114	10830808	0.75	-11	olfactory receptor 1701
Olr174	NM_001001028	10724415	0.62	-22	olfactory receptor 174
Olr176	NM_001000178	10724420	0.78	-16	olfactory receptor 176
Olr181	NM_001001030	10724426	0.71	-12	olfactory receptor 181
Olr190	NM_001000185	10709544	0.72	-15	olfactory receptor 190
Olr255	NM_001000947	10724672	0.70	-23	olfactory receptor 255
Olr323	NM_001000245	10728988	0.70	-12	olfactory receptor 323
Olr397	NM_001000380	10835839	0.77	-14	olfactory receptor 397
Olr408	NM_001000384	10835861	0.73	-10	olfactory receptor 408
Olr424	NM_001000392	10844768	0.73	-14	olfactory receptor 424
Olr425	NM_001000393	10844770	0.82	-16	olfactory receptor 425
Olr43	NM_001000541	10709366	0.70	-24	olfactory receptor 43
Olr434	NM_001000397	10835890	0.83	-13	olfactory receptor 434
Olr479	NM_001000305	10846946	0.79	-13	olfactory receptor 479
Olr556	NM_001000929	10837508	0.72	-19	olfactory receptor 556
Olr567	NM_001000326	10847060	0.74	-11	olfactory receptor 567
Olr57	NM_001000749	10724236	0.75	-15	olfactory receptor 57
Olr578	NM_001000568	10847072	0.73	-14	olfactory receptor 578
Olr60	NM_001000748	10709388	0.67	-19	olfactory receptor 60

Olr602	NM_001000333	10847082	0.76	-13	olfactory receptor 602
Olr637	NM_001001126	10837590	0.69	-14	olfactory receptor 637
Olr640	NM_001000644	10837594	0.74	-12	olfactory receptor 640
Olr652	NM_001000638	10847122	0.76	-11	olfactory receptor 652
Olr684	NM_001001378	10837624	0.76	-11	olfactory receptor 684
Olr728	NM_001000363	10837640	0.57	-23	olfactory receptor 728
Olr733	NM_001000618	10847240	0.59	-21	olfactory receptor 733
Olr734	NM_001000617	10847242	0.72	-14	olfactory receptor 734
Olr756	NM_001000614	10838414	0.61	-18	olfactory receptor 756
Olr767	NM_001000611	10848109	0.69	-13	olfactory receptor 767
Olr789	NM_001000377	10848144	0.72	-13	olfactory receptor 789
Olr803	NM_001000853	10862285	0.77	-12	olfactory receptor 803
Olr828	NM_001000991	10858150	0.69	-14	olfactory receptor 828
Olr839	NM_001000415	10876461	0.67	-23	olfactory receptor 839
Olr856	NM_001000515	10879321	0.74	-17	olfactory receptor 856
Olr86	NM_001000747	10709417	0.66	-20	olfactory receptor 86
**Olr862**	**NM_001001071**	**10879329**	**0.70**	**-16**	**olfactory receptor 862**
Olr865	NM_001000410	10879335	0.71	-16	olfactory receptor 865
Olr866	NM_001000411	10879337	0.44	-54	olfactory receptor 866
Olr868	NM_001000821	10879341	0.71	-22	olfactory receptor 868
Olr869	NM_001000413	10879332	0.53	-98	olfactory receptor 869
Olr881	NM_001000056	10899975	0.70	-10	olfactory receptor 881
Olr89	NM_001000745	10709424	0.74	-11	olfactory receptor 89
Olr907	NM_001001357	10893313	0.74	-13	olfactory receptor 907
Olr917	NM_001001354	10900006	0.75	-11	olfactory receptor 917
P2ry12	NM_022800	10823365	1.50	91	purinergic receptor P2Y, G-protein coupled, 12
Pgrmc2	NM_001008374	10822983	1.29	279	progesterone receptor membrane component 2
Pric285	ENSRNOT00000017787	10852492	0.81	-12	peroxisomal proliferator-activated receptor A interacting complex 285
Ptbp2	NM_001005555	10826418	0.81	-322	polypyrimidine tract binding protein 2
Rbp4	NM_013162	10729970	1.64	46	retinol binding protein 4, plasma
Ryr3	ENSRNOT00000049582	10848275	0.73	-12	ryanodine receptor 3
Sstr5	NM_012882	10741456	0.81	-11	somatostatin receptor 5
Taar7e	NM_175590	10717276	0.79	-15	trace-amine-associated receptor 7e
Tas2r114	NM_023996	10866312	0.70	-11	taste receptor, type 2, member 114
Tas2r126	NM_139335	10855079	0.77	-11	taste receptor, type 2, member 126
Tmem67	NM_001107916	10875537	0.79	-139	transmembrane protein 67
Topors	NM_001108658	10876007	1.31	273	topoisomerase I binding, arginine/serine-rich
Vom1r106	NM_001008965	10894293	0.69	-18	vomeronasal 1 receptor, 106
Vom1r29	NM_001008946	10703656	0.72	-10	vomeronasal 1 receptor, 29
Vom1r59	NM_001008944	10704301	0.76	-10	vomeronasal 1 receptor, 59
Vom1r63	NM_001008967	10812084	0.69	-21	vomeronasal 1 receptor, 63
Vom1r86	NM_001009527	10855840	0.67	-17	vomeronasal 1 receptor, 86
Vom1r87	NM_001009529	10855842	0.74	-13	vomeronasal 1 receptor, 87
**Vom2r3**	**NM_001099460**	**10701668**	**0.74**	**-41**	**vomeronasal 2 receptor, 3**
Vom2r59	NM_001099466	10756059	0.73	-12	vomeronasal 2 receptor, 59
**Signaling**					
Arhgap23	ENSRNOT00000055414	10737838	1.23	46	Rho GTPase activating protein 23
Defb33	NM_001037523	10792563	0.72	-12	defensin beta 33
Dusp6	NM_053883	10895144	1.41	567	dual specificity phosphatase 6
Gna13	NM_001013119	10748537	1.21	214	guanine nucleotide binding protein, alpha 13
Gper	NM_133573	10760655	1.26	11	G protein-coupled estrogen receptor 1
Mapkap1	NM_001011964	10835666	1.23	103	mitogen-activated protein kinase associated protein 1
Mobkl2a	NM_001108734	10893739	1.24	71	MOB1, Mps One Binder kinase activator-like 2A (yeast)
Mobkl2b	NM_001108970	10875954	0.83	-77	MOB1, Mps One Binder kinase

					activator-like 2B (yeast)
**Phospho2**	**NM_001007642**	**10836633**	**1.23**	**43**	**phosphatase, orphan 2**
Ppp1r14d	NM_172011	10848527	0.80	-16	protein phosphatase 1, regulatory (inhibitor) subunit 14D
Ppp1r1c	NM_001109200	10837195	1.59	35	protein phosphatase 1, regulatory (inhibitor) subunit 1C
Prkab1	NM_031976	10758940	1.28	153	protein kinase, AMP-activated, beta 1 non-catalytic subunit
Ptpdc1	NM_001106104	10794430	1.22	31	protein tyrosine phosphatase domain containing 1
Rab8b	NM_153317	10918374	1.26	489	RAB8B, member RAS oncogene family
Rasa3	NM_031574	10789401	1.30	57	RAS p21 protein activator 3
RGD1559699	ENSRNOT00000047604	10718167	0.44	-146	similar to putative protein kinase
**RGD1562638**	**NM_001100944**	**10788692**	**0.61**	**-20**	**similar to MAP/microtubule affinity-regulating kinase 3**
Rnd2	NM_001010953	10738423	1.28	125	Rho family GTPase 2
Sike	NM_001012182	10817910	1.33	145	suppressor of IKK epsilon
Smpdl3b	NM_001025737	10880339	0.79	-55	sphingomyelin phosphodiesterase, acid-like 3B
Sparcl1	NM_012946	10771210	1.59	122	SPARC-like 1 (hevin)
Strap	NM_001011969	10859371	1.23	300	serine/threonine kinase receptor associated protein
Wisp2	NM_031590	10842043	0.83	-13	WNT1 inducible signaling pathway protein 2
**Transcription**					
Ankrd10	ENSRNOT00000018870	10789560	0.83	-19	ankyrin repeat domain 10
Ascl1	NM_022384	10901531	2.50	161	achaete-scute complex homolog 1 (Drosophila)
Banp	NM_001106191	10808461	0.80	-121	Btg3 associated nuclear protein
Batf2	ENSRNOT00000028527	10713201	0.80	-10	basic leucine zipper transcription factor, ATF-like 2
Ccdc162	ENSRNOT00000000340	10833725	0.72	-16	coiled-coil domain containing 162
Ccdc32	NM_001024245	10848499	1.22	46	coiled-coil domain containing 32
Ccdc86	NM_001006974	10728830	1.22	113	coiled-coil domain containing 86
Dlx1	NM_001100531	10836841	1.25	22	distal-less homeobox 1
**Eid3**	**NM_001044304**	**10901436**	**1.29**	**55**	**EP300 interacting inhibitor of differentiation 3**
Etv3l	NM_001100992	10816303	0.71	-24	ets variant 3-like
Hand2	NM_022696	10791504	1.29	38	heart and neural crest derivatives expressed 2
Hivep2	NM_024137	10701830	1.27	70	human immunodeficiency virus type I enhancer binding protein 2
Jund	NM_138875	10787505	1.23	31	jun D proto-oncogene
**Klhl36**	**NM_001017511**	**10808356**	**1.36**	**52**	**kelch-like 36 (Drosophila)**
Med4	NM_001024256	10781443	1.50	185	mediator complex subunit 4
Narf	NM_001039207	10740240	1.22	84	nuclear prelamin A recognition factor
Obox2	ENSRNOT00000050817	10939183	0.77	-13	oocyte specific homeobox 2
Polq	NM_001105878	10754260	0.82	-38	polymerase (DNA directed), theta
RGD1564651	ENSRNOT00000059037	10703873	0.75	-13	similar to oocyte specific homeobox 3
Snai3	NM_001107439	10811581	0.82	-10	snail homolog 3 (Drosophila)
Trim24	NM_001044266	10861908	0.67	-38	tripartite motif-containing 24
Zfp9	NM_001127635	10864972	0.79	-20	zinc finger protein 9
Znrd1	NM_001166300	10827696	0.80	-195	zinc ribbon domain containing, 1
Hoxd12	NM_001191903	10836999	0.80	-11	homeo box D12
**Ddx25**	**NM_031630**	**10916060**	**1.23**	**36**	**DEAD (Asp-Glu-Ala-Asp) box polypeptide 25**
**Translation & Protein Modification**					
Tarsl2	NM_001014020	10707756	1.37	19	threonyl-tRNA synthetase-like 2
Rp9	NM_001108756	10915773	1.29	169	retinitis pigmentosa 9 (human)
Rsl24d1	NM_001014212	10911538	1.33	184	ribosomal L24 domain containing 1

Arl3	NM_022700	10730591	0.82	-102	ADP-ribosylation factor-like 3
Arl5a	NM_053979	10845298	1.26	209	ADP-ribosylation factor-like 5A
LOC683469	BC158599	10790603	0.71	-21	similar to RNA polymerase II transcription factor SIII subunit A2 (Elongin A2) (EloA2) (Transcription elongation factor B polypeptide 3B)
Mrps14	NM_001105963	10764983	1.23	130	mitochondrial ribosomal protein S14
RGD1561453	ENSRNOT00000049732	10714411	1.39	105	similar to ribosomal protein S12
RGD1564325	ENSRNOT00000047252	10790214	1.29	22	similar to ribosomal protein S24
Rnase4	NM_020082	10779832	1.51	46	ribonuclease, RNase A family 4
Arf6	NM_024152	10884961	1.25	617	ADP-ribosylation factor 6
Lsm5	NM_001107289	10862728	1.28	111	LSM5 homolog, U6 small nuclear RNA associated (S. cerevisiae)
Mrpl40	NM_001024865	10755987	1.22	105	mitochondrial ribosomal protein L40 (chr11_random)
Mrpl40	NM_001024865	10781424	1.23	109	mitochondrial ribosomal protein L40 (chr15)
Mrpl48	NM_001106282	10723982	1.23	163	mitochondrial ribosomal protein L48
Rbm41	NM_001109420	10939498	0.83	-30	RNA binding motif protein 41
Taf7	NM_001107394	10804015	1.20	510	TAF7 RNA polymerase II, TATA box binding protein (TBP)-associated factor, 55kDa
**Miscellaneous & Unknown**					
Fam43a	NM_001039002	10754939	1.26	50	family with sequence similarity 43, member A
**Fam50a**	**NM_001170573**	**10940358**	**0.83**	**-34**	**family with sequence similarity 50, member A**
LOC100137086	NM_001114656	10838959	0.66	-41	hypothetical protein LOC100137086
LOC100359800	ENSRNOT00000060303	10783474	0.78	-19	rCG64478-like
LOC100359861	ENSRNOT00000047282	10812903	1.23	293	mCG51409-like
LOC100360220	ENSRNOT00000060396	10780029	0.78	-22	rCG20053-like
LOC100360350	ENSRNOT00000060360	10779978	0.73	-16	mCG140239-like
LOC100361188	ENSRNOT00000021284	10859627	1.41	506	ghiso
LOC100361390	ENSRNOT00000060393	10780038	0.77	-11	mCG1025068-like
LOC100361418	ENSRNOT00000064258	10786126	0.76	-35	hypothetical protein LOC100361418
LOC100361589	ENSRNOT00000060344	10783416	0.73	-25	mCG140367-like
LOC100362690	ENSRNOT00000030635	10908998	0.54	-39	rCG64164-like
LOC100363541	ENSRNOT00000040239	10750108	0.54	-65	hypothetical protein LOC100363541
LOC100366148	ENSRNOT00000067743	10780082	0.75	-37	hypothetical protein LOC100366148
**LOC302845**	**NM_001013962**	**10935622**	**1.26**	**16**	**similar to mage-k1**
LOC366768	ENSRNOT00000051567	10892656	0.74	-37	mCG127631-like
LOC498759	ENSRNOT00000040660	10798621	0.79	-15	LRRGT00094
**LOC499234**	**ENSRNOT00000051854**	**10709667**	**0.71**	**-21**	**similar to NACHT, leucine rich repeat and PYD containing 14-like**
LOC678814	ENSRNOT00000060281	10780135	0.66	-32	hypothetical protein LOC678814
LOC680062	ENSRNOT00000066991	10779200	0.79	-92	hypothetical protein LOC680062
LOC682195	ENSRNOT00000057852	10766044	1.26	16	hypothetical protein LOC682195
LOC682999	NM_001127600	10766437	1.24	428	hypothetical protein LOC682999
LOC685105	ENSRNOT00000048639	10806501	0.61	-20	hypothetical protein LOC685105
LOC685261	XM_001063064	10752953	0.76	-34	hypothetical protein LOC685261
LOC685349	ENSRNOT00000061603	10750100	0.73	-37	hypothetical protein LOC685349
LOC685424	ENSRNOT00000050888	10809909	0.63	-42	hypothetical protein LOC685424
LOC685544	NM_001109474	10712457	0.70	-46	hypothetical protein LOC685544
LOC691011	ENSRNOT00000060368	10780088	0.76	-22	rCG38400-like
LOC691048	ENSRNOT00000068439	10783368	0.70	-18	RGD1359684 protein-like
LOC691079	ENSRNOT00000060356	10783439	0.81	-15	rCG64215-like
LOC691264	ENSRNOT00000048915	10780044	0.69	-22	rCG23598-like
RGD1305455	NM_001024969	10760877	1.26	101	similar to hypothetical protein FLJ10925
RGD1309106	NM_001014130	10769262	0.71	-15	similar to hypothetical protein (chr13)
RGD1310571	NM_001014147	10935033	1.31	281	similar to hypothetical protein (chrX)

RGD1560398	ENSRNOT00000056932	10759058	1.29	31	RGD1560398
RGD1562136	NM_001109086	10925753	1.35	63	similar to D1Ertd622e protein
RGD1562319	ENSRNOT00000060024	10760870	0.75	-17	similar to family with sequence similarity 55, member C
RGD1563569	ENSRNOT00000064359	10779984	0.76	-11	similar to variable region-alpha RAV4.22
RGD1564195	ENSRNOT00000049044	10725914	0.73	-28	similar to hypothetical protein
RGD1565033	NM_001109050	10739206	1.29	72	similar to hypothetical protein LOC284018 isoform b
RGD1565493	XM_002729803	10903288	0.71	-17	similar to DKFZP434I092 protein
Rwdd4a	NM_001034994	10938439	1.35	165	RWD domain containing 4A
Samd12	NM_001130562	10903721	0.78	-13	sterile alpha motif domain containing 12
Sgp158	NM_139184	10859183	0.72	-16	proline-rich glycoprotein (sgp158)
Spetex-2D	NM_001011701	10779288	0.56	-69	Spetex-2D protein
**Tdrd12**	**ENSRNOT00000017021**	**10721099**	**1.31**	**17**	**tudor domain containing 12**
Tmco6	NM_001106154	10801074	0.81	-84	transmembrane and coiled-coil domains 6
Ttc19	NM_001109644	10734294	1.37	91	tetratricopeptide repeat domain 19
**Wdr46**	**NM_212491**	**10831747**	**1.24**	**147**	**WD repeat domain 46**
**microRNA**					
Mir135a	NR_031881	10901840	0.68	-13	microRNA mir-135a
Mir17-1	NR_031818	10781974	0.78	-14	microRNA mir-17-1
Mir214	NR_031927	10765111	0.75	-44	microRNA mir-214
Mir215	NR_032110	10766510	0.68	-14	microRNA mir-215
Mir296	NR_031940	10852170	0.72	-18	microRNA mir-296
Mir330	NR_031777	10704784	0.78	-17	microRNA mir-330
Mir98	NR_031855	10933128	0.80	-28	microRNA mir-98
Mirlet7b	NR_031802	10898357	0.81	-18	microRNA let-7b
Mir218-1	NR_031931	10777176	0.58	-138	microRNA mir-218-1
Mir341	rno-mir-341	10886842	0.78	-56	microRNA mir-341
Mir346	NR_031790	10787029	0.81	-91	microRNA mir-346
**EST's**					
LOC290071	BC088274	10780026	0.69	-35	similar to RIKEN cDNA A430107P09 gene
RGD1311273	NM_001109475	10741629	1.30	37	similar to RIKEN cDNA 9530058B02
RGD1305207	BC079419	10899416	0.80	-22	similar to RIKEN cDNA 1700011A15
RGD1561557	NM_001108929	10829351	0.79	-83	similar to chromosome 21 open reading frame 29
RGD1309374	ENSRNOT00000020492	10703327	0.83	-61	LOC361485
RGD1305939	NM_001130581	10897648	0.67	-23	hypothetical LOC300074
RGD1306636	ENSRNOT00000055016	10725887	0.80	-13	hypothetical LOC293498
	AF254801	10757629	0.83	-13	
	ENSRNOT00000002781	10891491	0.66	-15	
	ENSRNOT00000004499	10833361	1.26	24	
	ENSRNOT00000005636	10904230	0.78	-17	
	ENSRNOT00000006775	10884898	1.52	93	
	ENSRNOT00000007380	10766078	0.72	-32	
	ENSRNOT00000007844	10890949	0.74	-13	
	ENSRNOT00000008848	10862850	1.23	55	
	ENSRNOT00000009816	10852733	0.82	-34	
	ENSRNOT00000012507	10824763	0.79	-40	
	ENSRNOT00000013436	10704380	1.84	127	
	ENSRNOT00000016081	10792132	1.36	128	
	ENSRNOT00000017015	10854357	1.20	20	
	ENSRNOT00000018039	10923336	1.44	81	
	ENSRNOT00000024332	10850988	1.24	47	
	ENSRNOT00000024516	10729852	1.25	220	
	ENSRNOT00000024688	10793706	1.21	10	
	ENSRNOT00000024998	10709364	0.79	-19	
	ENSRNOT00000025383	10920986	0.70	-72	
	ENSRNOT00000029032	10787958	1.29	23	
	ENSRNOT00000030299	10782761	0.71	-16	

	**ENSRNOT00000030811**	**10770140**	**1.48**	**40**	
	ENSRNOT00000031816	10894159	1.24	111	
	ENSRNOT00000032578	10918485	0.81	-24	
	**ENSRNOT00000032989**	**10938820**	**0.73**	**-16**	
	ENSRNOT00000033247	10756127	0.81	-28	
	ENSRNOT00000033434	10939180	0.83	-44	
	ENSRNOT00000034281	10771765	1.37	161	
	ENSRNOT00000034419	10833894	1.37	27	
	ENSRNOT00000034550	10779334	0.77	-16	
	ENSRNOT00000034660	10806869	0.73	-20	
	ENSRNOT00000034855	10795361	0.67	-17	
	ENSRNOT00000035375	10820096	1.35	21	
	ENSRNOT00000035387	10934046	0.77	-26	
	ENSRNOT00000036265	10845363	1.27	26	
	ENSRNOT00000036335	10835888	0.72	-12	
	**ENSRNOT00000036735**	**10854239**	**1.35**	**37**	
	ENSRNOT00000036884	10795677	1.40	28	
	ENSRNOT00000038163	10901036	1.39	283	
	ENSRNOT00000038381	10742118	1.33	20	
	ENSRNOT00000038522	10902012	0.76	-20	
	ENSRNOT00000039834	10732924	1.48	31	
	ENSRNOT00000039879	10897891	1.29	78	
	ENSRNOT00000039948	10767044	1.45	883	
	ENSRNOT00000039983	10747141	0.78	-67	
	ENSRNOT00000040043	10915273	0.59	-20	
	ENSRNOT00000040554	10718155	0.72	-30	
	ENSRNOT00000040940	10904601	1.39	44	
	ENSRNOT00000042573	10879913	0.72	-16	
	ENSRNOT00000042949	10839417	1.30	15	
	ENSRNOT00000043132	10720555	0.49	-304	
	ENSRNOT00000043225	10933166	1.25	24	
	ENSRNOT00000044403	10775741	0.76	-12	
	ENSRNOT00000044643	10717398	0.74	-20	
	ENSRNOT00000044695	10862982	0.81	-12	
	ENSRNOT00000046555	10892346	0.79	-89	
	ENSRNOT00000046658	10924505	0.66	-97	
	ENSRNOT00000047283	10827409	0.80	-16	
	**ENSRNOT00000047567**	**10842660**	**1.21**	**16**	
	ENSRNOT00000047758	10775226	0.69	-70	
	ENSRNOT00000047949	10704169	0.70	-11	
	ENSRNOT00000048037	10892490	0.68	-22	
	ENSRNOT00000048310	10802601	0.82	-13	
	ENSRNOT00000048840	10747160	0.80	-63	
	ENSRNOT00000048906	10800328	0.80	-12	
	ENSRNOT00000049343	10938547	0.61	-30	
	ENSRNOT00000050510	10724593	0.58	-48	
	ENSRNOT00000051223	10791274	0.60	-139	
	ENSRNOT00000051410	10892671	0.51	-32	
	ENSRNOT00000051657	10780085	0.75	-23	
	ENSRNOT00000051864	10719071	0.73	-18	
	ENSRNOT00000052478	10871052	0.62	-33	
	ENSRNOT00000052538	10819400	0.82	-12	
	ENSRNOT00000052877	10820010	0.75	-14	
	ENSRNOT00000052950	10819944	0.69	-51	
	ENSRNOT00000052962	10845441	0.77	-17	
	ENSRNOT00000052993	10722475	0.65	-21	
	ENSRNOT00000053060	10822390	0.57	-26	
	ENSRNOT00000053081	10722479	0.58	-40	
	ENSRNOT00000053175	10804243	0.80	-75	
	ENSRNOT00000053205	10701988	0.77	-14	
	ENSRNOT00000053262	10925572	0.80	-37	
	ENSRNOT00000053285	10816263	0.76	-12	
	**ENSRNOT00000053306**	**10854959**	**0.72**	**-24**	

	ENSRNOT00000053496	10903998	0.75	-38	
	ENSRNOT00000053524	10850666	0.78	-41	
	ENSRNOT00000053867	10805075	0.63	-44	
	ENSRNOT00000053929	10839872	0.80	-948	
	ENSRNOT00000053958	10722401	0.65	-18	
	ENSRNOT00000054191	10807031	0.52	-24	
	ENSRNOT00000054348	10787050	0.71	-22	
	ENSRNOT00000054515	10794274	0.83	-11	
	ENSRNOT00000054567	10856162	0.74	-40	
	ENSRNOT00000055140	10738944	0.72	-23	
	ENSRNOT00000056771	10892648	0.79	-12	
	ENSRNOT00000056835	10892478	0.79	-11	
	ENSRNOT00000056841	10879107	0.80	-66	
	ENSRNOT00000056846	10939615	0.69	-97	
	ENSRNOT00000056866	10857468	0.76	-13	
	ENSRNOT00000057274	10911568	0.82	-15	
	ENSRNOT00000057494	10863683	0.73	-19	
	ENSRNOT00000057576	10773797	0.77	-26	
	ENSRNOT00000057905	10846668	0.71	-64	
	ENSRNOT00000058202	10895665	0.73	-21	
	ENSRNOT00000058407	10895524	0.75	-13	
	ENSRNOT00000058693	10781288	1.33	90	
	ENSRNOT00000058857	10890599	0.80	-11	
	ENSRNOT00000059064	10781110	0.78	-16	
	ENSRNOT00000059332	10764974	0.77	-31	
	ENSRNOT00000059528	10718428	0.72	-16	
	ENSRNOT00000059665	10836062	0.79	-14	
	ENSRNOT00000059747	10732800	0.43	-151	
	ENSRNOT00000059976	10801592	0.75	-46	
	ENSRNOT00000060162	10776211	0.72	-209	
	ENSRNOT00000060259	10876463	0.78	-59	
	ENSRNOT00000060285	10783521	0.76	-55	
	ENSRNOT00000060299	10783495	0.77	-21	
	ENSRNOT00000060660	10794310	0.79	-10	
	ENSRNOT00000060749	10908655	0.70	-108	
	ENSRNOT00000060815	10717588	0.54	-261	
	ENSRNOT00000061137	10717389	0.81	-18	
	ENSRNOT00000062081	10834071	0.73	-84	
	ENSRNOT00000062990	10722513	0.65	-44	
	ENSRNOT00000063480	10860865	0.76	-23	
	ENSRNOT00000063489	10884472	0.67	-20	
	ENSRNOT00000063607	10867526	0.77	-13	
	ENSRNOT00000063767	10715308	0.61	-16	
	ENSRNOT00000066333	10913991	1.28	29	
	**ENSRNOT00000068960**	**10814096**	**0.58**	**-22**	
	ENSRNOT00000069084	10723553	0.66	-13	
	ENSRNOT00000069176	10811704	0.78	-13	
	ENSRNOT00000069806	10776287	0.78	-17	
	ENSRNOT00000070467	10792022	0.77	-11	
	FQ220821	10831802	1.34	50	
	FQ223635	10797821	1.25	181	
	**FQ224515**	**10823593**	**1.40**	**84**	
	FQ228224	10856191	0.78	-12	
	FQ232545	10794320	1.27	164	
	GENSCAN00000006519	10751341	1.24	33	
	GENSCAN00000009330	10916917	0.77	-33	
	GENSCAN00000011788	10940007	1.25	48	
	GENSCAN00000015898	10763457	0.72	-15	
	GENSCAN00000028416	10752259	0.57	-25	
	GENSCAN00000034118	10938647	1.29	34	
	**NC_001665**	**10930620**	**1.44**	**524**	
	---	10725041	0.71	-59	
	---	10707118	0.77	-19	

	---	10710338	0.65	-13	
	---	10724387	0.63	-13	
	---	10732056	0.73	-20	
	---	10737045	0.77	-17	
	---	10750511	0.61	-31	
	---	10750023	0.64	-22	
	---	10758894	0.82	-13	
	---	10764142	0.80	-10	
	---	10772909	0.75	-13	
	---	10792099	0.61	-20	
	---	10790615	0.76	-15	
	---	10803841	0.71	-374	
	---	10809090	0.44	-27	
	---	10825573	0.73	-55	
	---	10826983	0.66	-17	
	---	10844687	0.63	-75	
	---	10843137	1.59	155	
	---	10845605	1.22	247	
	---	10864556	0.81	-16	
	---	10857067	0.80	-11	
	---	10872129	0.68	-74	
	---	10874979	0.76	-43	
	---	10868550	0.69	-21	
	---	10916853	0.80	-55	
	---	10908746	0.67	-34	

25 Genes overlapped with E16 PGC list are marked by bold font.
