# Supplementary material for: Environmentally Induced Transgenerational Epigenetic Reprogramming of Primordial Germ Cells and the Subsequent Germ Line
Source: PLoS One. 2013 Jul 15;8(7):e66318. doi: 10.1371/journal.pone.0066318 (PMC3712023; doi:10.1371/journal.pone.0066318)
Supplement: Table S2 — Genes differentially expressed in E16 F3 generation germ cells. The 25 genes that were also found among genes differentially expressed in F3 generation PGCs at E13 are marked by bold font. (PDF) [file pone.0066318.s003.pdf]

Supplemental Table S2

Genes Differentially Expressed in E16 F3 Generation Germ Cells

**GENE FUNCTIONAL GROUP/** Gene Symbol	**RefSeq/GenBank/ Ensemble Number**	**Affymetrix Probset ID**	**Vin/Con**	**Mean Diff.**	**Gene Title**
**Apoptosis**					
Apaf1	NM_023979	10901783	0.83	-86	apoptotic peptidase activating factor 1
**Cell Cycle**					
Cep135	ENSRNOT00000032156	10776370	0.81	-23	centrosomal protein 135kDa
Ddit3	NM_001109986	10895861	0.74	-48	DNA-damage inducible transcript 3
Rprd1b	NM_001098727	10841669	0.79	-42	regulation of nuclear pre-mRNA domain containing 1B
**Cytoskeleton-ECM**					
LOC687234	ENSRNOT00000002004	10761245	0.79	-50	similar to actin related protein 2/3 complex, subunit 5
Cadm1	NM_001012201	10909761	0.80	-62	cell adhesion molecule 1
Cgn	ENSRNOT00000028440	10824860	0.73	-60	cingulin
Dnah11	ENSRNOT00000007233	10892706	0.83	-19	dynein, axonemal, heavy chain 11
Mcoln1	NM_001105903	10759698	1.21	295	mucolipin 1
**Pcdh15**	**ENSRNOT00000066040**	**10832646**	**1.22**	**12**	**protocadherin 15**
**Development**					
Cpne3	NM_001107917	10875680	0.76	-283	copine III
Gtsf1	NM_001079707	10907722	1.41	154	gametocyte specific factor 1
Lin37	NM_001106245	10720648	1.25	154	lin-37 homolog (C. elegans)
**LOC171573**	**NM_138537**	**10909012**	**1.24**	**47**	**spleen protein 1 precursor**
LOC685411	ENSRNOT00000041115	10806538	1.40	12	similar to spermatogenesis associated glutamate (E)-rich protein 4b
Meig1	NM_001134882	10796413	0.70	-24	meiosis expressed gene 1
**Mospd1**	**NM_001014107**	**10939816**	**0.82**	**-41**	**motile sperm domain containing 1**
Mospd2	NM_001134588	10933408	0.75	-143	motile sperm domain containing 2
Shank1	NM_031751	10706515	1.24	17	SH3 and multiple ankyrin repeat domains 1
Tmem231	ENSRNOT00000031243	10811208	0.81	-81	transmembrane protein 231
Trank1	NM_001191799	10913866	0.77	-529	tetratricopeptide repeat and ankyrin repeat containing 1
**Electron Transport**					
Cyp20a1	NM_199401	10923768	0.83	-58	cytochrome P450, family 20, subfamily a, polypeptide 1
**Epigenetics**					
Hdac8	NM_001126373	10938734	1.23	22	histone deacetylase 8
Hfm1	NM_001191101	10771099	1.28	27	HFM1, ATP-dependent DNA helicase homolog (S. cerevisiae)
**Golgi Apparatus**					
LOC685079	ENSRNOT00000030054	10842117	1.37	180	similar to Protein SYS1 homolog
**Growth Factors & Hormones**					
Cxcr7	NM_053352	10925291	0.69	-226	chemokine (C-X-C motif) receptor 7
Adipoq	NM_144744	10755112	1.28	21	adiponectin, C1Q and collagen domain containing
**Immune Response**					
Cd3d	NM_013169	10909583	1.22	11	CD3 molecule delta polypeptide
RGD1305090	NM_001162535	10879734	1.35	34	similar to CD2-associated protein
Sart1	NM_031596	10713040	1.22	19	squamous cell carcinoma antigen recognized by T cells
Toag1	NM_001110838	10914442	0.82	-36	tolerance-associated gene 1
**Metabolism & Transport**					
Gls2	NM_138904	10893008	1.20	31	glutaminase 2 (liver, mitochondrial)
**Hmox1**	**NM_012580**	**10806122**	**0.73**	**-69**	**heme oxygenase (decycling) 1**
Kctd18	NM_001106914	10928220	1.22	83	potassium channel tetramerisation

					domain containing 18
Kctd21	NM_001109151	10723718	1.26	15	potassium channel tetramerisation domain containing 21
Pgd	ENSRNOT00000018401	10881669	0.80	-52	phosphogluconate dehydrogenase
Pla2g2c	NM_019202	10873327	1.33	18	phospholipase A2, group IIC
Sdr39u1	NM_001108378	10783981	1.25	112	short chain dehydrogenase/reductase family 39U, member 1
Slc16a14	NM_001108229	10929536	0.77	-21	solute carrier family 16, member 14 (monocarboxylic acid transporter 14)
**Slc25a25**	**NM_145677**	**10844339**	**0.83**	**-20**	**solute carrier family 25 (mitochondrial carrier, phosphate carrier), member 25**
Slc35f2	NM_001106822	10910038	1.31	17	solute carrier family 35, member F2
Slc41a2	NM_001108742	10894606	1.31	112	solute carrier family 41, member 2
Sult2b1	NM_001039665	10721892	1.24	70	sulfotransferase family, cytosolic, 2B, member 1
Timm8a2	NM_001109429	10782124	1.26	159	translocase of inner mitochondrial membrane 8 homolog a2 (yeast)
Tmed5	NM_001007619	10771004	0.80	-57	transmembrane emp24 protein transport domain containing 5
**Proteolysis**					
Cst13	NM_001109343	10840597	0.75	-30	cystatin 13
Gzma	NM_153468	10821370	1.78	124	granzyme A
Ky	NM_001108180	10912567	1.29	18	kyphoscoliosis peptidase
Rnf151	NM_001106987	10741235	0.81	-28	ring finger protein 151
**Receptors & Binding Proteins**					
Fabp9	NM_022854	10814294	1.29	20	fatty acid binding protein 9, testis
Hhipl1	ENSRNOT00000034883	10886728	1.27	28	HHIP-like 1
Olr1435	NM_001000777	10742952	1.26	10.2	olfactory receptor 1435
Olr1619	NM_001000521	10779782	0.75	-16	olfactory receptor 1619
**Olr862**	**NM_001001071**	**10879329**	**1.33**	**11**	**olfactory receptor 862**
**Vom2r3**	**NM_001099460**	**10701643**	**0.77**	**-14**	**vomeronasal 2 receptor, 3**
**Signaling**					
Ctnnal1	NM_001106649	10876896	0.78	-121	catenin (cadherin associated protein), alpha-like 1
Dhh	NM_053367	10907056	0.71	-104	desert hedgehog homolog (Drosophila)
Gch1	NM_024356	10782919	0.80	-13	GTP cyclohydrolase 1
Hipk1	NM_001100986	10825580	0.73	-116	homeodomain interacting protein kinase 1
LOC679596	ENSRNOT00000047078	10812017	1.21	15	similar to GABA(A) receptor-associated protein like 2
Mlst8	NM_022404	10732079	0.82	-24	MTOR associated protein, LST8 homolog (S. cerevisiae)
Nkap	NM_001024872	10936289	0.71	-70	NFKB activating protein
**Phospho2**	**NM_001007642**	**10836633**	**1.32**	**108**	**phosphatase, orphan 2**
Plk2	NM_031821	10812954	0.73	-156	polo-like kinase 2 (Drosophila)
Prrg4	NM_001109203	10847957	0.74	-122	proline rich Gla (G-carboxyglutamic acid) 4 (transmembrane)
Rab3a	NM_013018	10790912	1.23	177	RAB3A, member RAS oncogene family
Rab3il1	NM_134411	10713833	0.80	-31	RAB3A interacting protein (rabin3)-like 1
**RGD1562638**	**NM_001100944**	**10788692**	**1.44**	**14**	**similar to MAP/microtubule affinity-regulating kinase 3**
Upk3b	ENSRNOT00000037639	10761101	1.35	14	uroplakin 3B
**Transcription**					
Bhlhe41	AF009329	10867026	1.40	41	basic helix-loop-helix family, member e41
Ccdc106	ENSRNOT00000021613	10718817	1.26	45	coiled-coil domain containing 106
Ccdc130	NM_001037644	10806687	1.25	148	coiled-coil domain containing 130
Ccdc163	NM_001025656	10871293	0.78	-24	coiled-coil domain containing 163
**Ddx25**	**NM_031630**	**10916060**	**1.30**	**137**	**DEAD (Asp-Glu-Ala-Asp) box polypeptide 25**
Dhx32	NM_001130039	10726358	1.23	92	DEAH (Asp-Glu-Ala-His) box polypeptide 32

**Eid3**	**NM_001044304**	**10901436**	**0.83**	**-45**	**EP300 interacting inhibitor of differentiation 3**
Fbxl20	NM_022272	10746856	1.21	207	F-box and leucine-rich repeat protein 20
Fbxo30	NM_001007690	10701788	0.82	-142	F-box protein 30
**Klhl36**	**NM_001017511**	**10808356**	**0.75**	**-108**	**kelch-like 36 (Drosophila)**
Ndrg4	NM_031967	10809100	1.44	51	N-myc downstream regulated gene 4
Nobox	NM_001192013	10862317	1.35	207	NOBOX oogenesis homeobox
Phf7	NM_001012211	10790119	1.21	38	PHD finger protein 7
Polr2d	NM_001108886	10800667	1.20	202	polymerase (RNA) II (DNA directed) polypeptide D
Zc3h13	NM_001170471	10780964	0.82	-107	zinc finger CCCH type containing 13
Zranb2	NM_031616	10819890	0.83	-297	zinc finger, RAN-binding domain containing 2
**Translation & Protein Modification**					
Arl5b	NM_001015031	10796543	0.79	-191	ADP-ribosylation factor-like 5B
Cabc1	NM_001013185	10770313	1.21	115	chaperone, ABC1 activity of bc1 complex homolog (S. pombe)
Trmt12	NM_001122976	10896745	1.22	11	tRNA methyltransferase 12 homolog (S. cerevisiae)
**Miscellaneous & Unknown**					
Fam178b	NM_001122658	10927402	1.42	381	family with sequence similarity 178, member B
Fam38a	NM_001077200	10811596	0.83	-35	family with sequence similarity 38, member A
**Fam50a**	**NM_001170573**	**10856453**	**0.81**	**-37**	**family with sequence similarity 50, member A**
LOC100365542	XM_002725468	10717350	1.33	19	rCG41957-like
LOC100366112	ENSRNOT00000036989	10934470	1.39	34	rCG64283-like
**LOC302845**	**NM_001013962**	**10935622**	**0.83**	**-40**	**similar to mage-k1**
**LOC499234**	**ENSRNOT00000051854**	**10709667**	**1.37**	**112**	**similar to NACHT, leucine rich repeat and PYD containing 14-like**
LOC500625	ENSRNOT00000006877	10883445	0.82	-87	hypothetical protein LOC500625
LOC502822	ENSRNOT00000040099	10863026	1.30	14	mCG130744-like
RGD1308350	ENSRNOT00000018225	10887486	1.32	43	similar to hypothetical protein MGC13251
RGD1565119	ENSRNOT00000048106	10776873	0.79	-28	similar to Mitochondrial carrier triple repeat 1
Rsb66	NM_181694	10844055	0.82	-13	Rsb-66 protein
Samd5	NM_001108901	10716704	0.77	-10	sterile alpha motif domain containing 5
**Tdrd12**	**ENSRNOT00000017021**	**10721099**	**1.49**	**110**	**tudor domain containing 12**
Tmem116	NM_001159625	10762108	1.42	49	transmembrane protein 116
Tmem135	NM_001013896	10723560	0.79	-109	transmembrane protein 135
**Wdr46**	**NM_212491**	**10831747**	**1.26**	**293**	**WD repeat domain 46**
Wdr53	NM_001109055	10754862	1.23	35	WD repeat domain 53
**microRNA**					
Mir19a	NR_031822	10781978	0.62	-136	microRNA mir-19a
Mir20a	NR_031793	10781980	0.74	-51	microRNA mir-20a
**EST's**					
LOC691286	NM_001109634	10742913	1.22	13	similar to RIKEN cDNA 4930504O13
RGD1309482	NM_001014246	10729979	0.72	-69	similar to chromosome 10 open reading frame 4; similar to putative acid phosphatase F26C11.1
RGD1311078	BC168942	10739781	1.22	268	LOC360664
	---	10742386	0.82	-257	
	---	10934982	0.71	-18	
	---	10903594	1.20	10.4	
	ENSRNOT00000006064	10902791	0.79	-19	
	ENSRNOT00000008360	10893453	1.26	38	
	ENSRNOT00000029207	10821047	1.25	45	
	**ENSRNOT00000030811**	**10770140**	**0.78**	**-27**	
	ENSRNOT00000031638	10772802	0.79	-16	
	**ENSRNOT00000032989**	**10938820**	**1.28**	**20**	
	**ENSRNOT00000036735**	**10854239**	**0.75**	**-81**	

	ENSRNOT00000037885	10885903	1.25	29	
	ENSRNOT00000041436	10869874	1.27	13	
	ENSRNOT00000041963	10938531	1.23	28	
	ENSRNOT00000047129	10716982	1.23	19	(chr1)
	ENSRNOT00000047129	10902883	1.23	19	(chr7)
	ENSRNOT00000047129	10918829	1.23	19	(chr8)
	ENSRNOT00000047129	10836210	1.25	21	(chr3)
	**ENSRNOT00000047567**	**10842660**	**0.83**	**-32**	
	ENSRNOT00000051759	10915740	0.79	-48	
	ENSRNOT00000052060	10783533	1.26	49	
	ENSRNOT00000052472	10728561	0.46	-1953	
	**ENSRNOT00000053306**	**10854959**	**1.23**	**15**	
	ENSRNOT00000053925	10755670	1.35	94	
	ENSRNOT00000054149	10734340	0.72	-337	
	ENSRNOT00000054274	10885448	0.72	-27	
	ENSRNOT00000057118	10833811	0.80	-550	
	ENSRNOT00000057159	10766878	1.24	11	
	ENSRNOT00000059351	10822320	1.28	11	
	ENSRNOT00000059648	10854733	1.31	14	
	**ENSRNOT00000068960**	**10904356**	**1.30**	**29**	
	FQ212434	10905587	0.82	-69	
	**FQ224515**	**10823593**	**0.82**	**-51**	
	**NC_001665**	**10930612**	**1.36**	**1119**	

25 Genes overlapped with E13 PGC list are marked by bold font.
